# The impact of different immunosuppressants and acute immune rejection on clinical outcomes in diverse solid organ transplant recipients

**DOI:** 10.3389/fimmu.2025.1739468

**Published:** 2026-01-30

**Authors:** Zhihao Wang, Zhenyu Liu, Xia Wu, Xiong Zeng, Tong Zhang, Ziqiang Li

**Affiliations:** 1 Organ Transplantation Clinical Medical Center of Xiamen University, Department of General Surgery, Xiang’an Hospital of Xiamen University, School of Medicine, Xiamen University, Xiamen, China; 2 Organ Transplantation Institute of Xiamen University, Xiamen Human Organ Transplantation Quality Control Center, Xiamen Key Laboratory of Regeneration Medicine, Fujian Provincial Key Laboratory of Organ and Tissue Regeneration, School of Medicine, Xiamen University, Xiamen, China; 3 Department of Liver Transplantation and Hepatic Surgery, the First Affiliated Hospital of Shandong First Medical University and Shandong Provincial Qianfoshan Hospital, Jinan, China

**Keywords:** acute immune rejection(AR), immunosuppressants, patient survival, solid organ transplantation (SOT), SRTR database

## Abstract

The success of solid organ transplantation (SOT) and the use of immunosuppressants provide patients with terminal conditions hope. Acute immune rejection (AR) in SOT patients, however, has become more noticeable. Our study examined the relationship between AR and patient survival in a variety of organ transplants, including liver, kidney, heart, lung, pancreas, intestine, combined heart–lung, and pancreas–kidney transplantations, using the Scientific Registry of Transplant Recipients (SRTR) database. Our research showed that AR universally reduces survival across all solid organ transplant types. Immunosuppressants exhibit organ-specific efficacy patterns, with divergent impacts on survival and AR risk. For instance, in liver transplants (LT), generic tacrolimus increased AR risk (OR: 1.31; 95% CI: 1.21–1.42), while AZA reduced it (OR: 0.52; 95% CI: 0.44–0.60). In kidney transplants (KT), tacrolimus increased AR risk (OR: 1.24; 95% CI: 1.2–1.28), whereas Cyclosporin reduced it (OR: 0.47; 95% CI: 0.43–0.52). Furthermore, the same immunosuppressant can have varying effects on survival across transplant types; MMF significantly increased the risk of death in LT, HT, LU, KT, HL, and PK patients, but reduced the risk of death in PT patients. Originator and generic immunosuppressants differentially influence survival outcomes and rejection incidence. For example, in heart transplantation (HT), originator cyclosporine improved survival, while generic cyclosporine (EON) was associated with decreased survival and increased AR risk. Overall, our research offers a thorough and methodical evaluation of how various immunosuppressants affect prognosis and how AR affects the survival of patients receiving different kinds of SOT.

## 1 Introduction

Remarkable advancements in solid organ transplantation (SOT) have brought renewed hope to patients with end-stage disease, but its clinical success has fundamentally depended on the rational application of immunosuppressants (1–5). However, the intricacy of immunosuppressive therapy lies in not only balancing graft survival with drug toxicity but also addressing the core challenge of post transplantation immune rejection. As a predominant contributor to graft dysfunction/loss, transplant rejection involves multiple mechanisms, including T-cell-mediated AR, antibody-mediated chronic rejection, and the activation of immune memory cells (6–9). Although immunosuppressants centered on calcineurin inhibitors (CNIs), mammalian target of rapamycin (mTOR) inhibitors, and antiproliferative agents have significantly reduced rejection rates, substantial therapeutic heterogeneity persists across drug categories and formulations—particularly between originators and generics—regarding therapeutic efficacy, safety profiles, and long-term prognostic impacts (10). Immunosuppressants are designed to reduce rejection risk; however, due to their pharmacokinetic properties, toxicity, and individual patient variability, they may indirectly increase rejection risk through factors such as metabolic genetic polymorphisms (e.g., CYP3A5 enzyme activity), infection-induced immune imbalance, metabolic disturbances, suboptimal dosing regimens, inadequate dose adjustments, or treatment interruption (11–16). For example, while CNIs remain first-line agents, their dose-dependent nephrotoxicity may accelerate the development of graft dysfunction; conversely, mTOR inhibitors demonstrate antineoplastic and antifibrotic potential yet are associated with elevated risks of proteinuria and impaired wound healing. Furthermore, although originator immunosuppressants and their generic counterparts meet bioequivalence standards, their clinical disparities in nonequivalence parameters, including pharmaceutical stability and immunosuppressive intensity, remain a matter of debate regarding their impacts on rejection control and long-term graft/patient survival (17, 18).

Notably, the classification of immune rejection and individualized management strategies remains contentious. While AR can be promptly managed through intensified immunosuppressants, chronic rejection, characterized by its multifactorial pathogenesis and limited therapeutic options, remains the primary barrier to long-term allograft survival (19–21). Emerging evidence suggests that select immunosuppressants (e.g., belatacept) or biologics (e.g., rituximab) may improve outcomes in antibody-mediated rejection (AMR) through targeted modulation of costimulatory pathways or B-cell depletion (22–24). However, systematic evaluation of the efficacy heterogeneity of these immunosuppressants across transplant types (renal *vs.* hepatic *vs.* cardiac allografts) is required to establish organ-specific therapeutic paradigms (25–27).

This investigation, in which the Scientific Registry of Transplant Recipients (SRTR) database was leveraged, addresses four pivotal questions: 1) Post transplantation AR adversely affects the survival of patients with liver, kidney, heart, lung, pancreas, heart-lung, pancreas-kidney and intestine transplant.; 2) The effects of the same immunosuppressant on overall survival varied across different types of solid organ transplant populations; 3) The effects of the same immunosuppressant on the development of post transplantation acute immune rejection also showed inconsistency across different solid organ transplant populations; 4) Originator and generic immunosuppressants have different effects on the prognosis and AR in solid organ transplant patients. Through multicenter retrospective cohort analysis and survival modeling, this study establishes evidence-based guidance for personalized immunosuppressive management and rejection mitigation, ultimately aiming to improve long-term clinical outcomes in transplant recipients.

## 2 Patients and methods

### 2.1 Data sources

Data from the Scientific Registry of Transplant Recipients (SRTR) database (https://www.srtr.org/) were utilized in this study to investigate patients undergoing various transplantation procedures. As a widely recognized resource in solid organ transplantation (SOT) research, the SRTR database served as the primary data source for this investigation.

### 2.2 Definitions

AR was defined according to the diagnostic criteria established by the Transplantation Society. Corticosteroids included prednisone, methylprednisolone (Solu-Medrol), and Medrol. Calcineurin inhibitors (CNIs) included tacrolimus (Prograf), cyclosporine formulations (Neoral, Sandimmune, and Gengraf), extended-release tacrolimus (Astagraf XL), and generic equivalents (generic Prograf for tacrolimus and EON for cyclosporine). mTOR inhibitors included sirolimus (Rapamune) and everolimus (Zortress). Antiproliferative agents consisted of azathioprine (AZA), mycophenolate mofetil (MMF; CellCept), mycophenolic acid (Myfortic), and generic MMF (generic CellCept). Polyclonal/monoclonal antibodies included muromonab-CD3 (OKT3), basiliximab (Simulect), alemtuzumab (Campath), rituximab (Rituxan), daclizumab (Zenapax), antilymphocyte globulin (ALG), thymoglobulin and Atgam. Alkylating agents included cyclophosphamide (Cytoxan). The novel immunosuppressants used were FTY720 (fingolimod) and belatacept (Nulojix).

### 2.3 Statistical analysis

Survival analysis was performed via the Kaplan–Meier method with log-rank tests. Cox proportional hazards regression models were employed to assess the impact of various immunosuppressants on transplant recipient survival, generating hazard ratios (HRs) with 95% confidence intervals (CIs). Logistic regression analysis was used to evaluate the associations between immunosuppressants and AR risk, and odds ratios (ORs) with 95% CIs were reported. Statistical computations were primarily conducted using RStudio version 4.4 (https://www.rstudio.com/). A significance threshold of P < 0.05 was applied for all inferential analyses.

## 3 Results

### 3.1 Patient population

**Figure 1:**
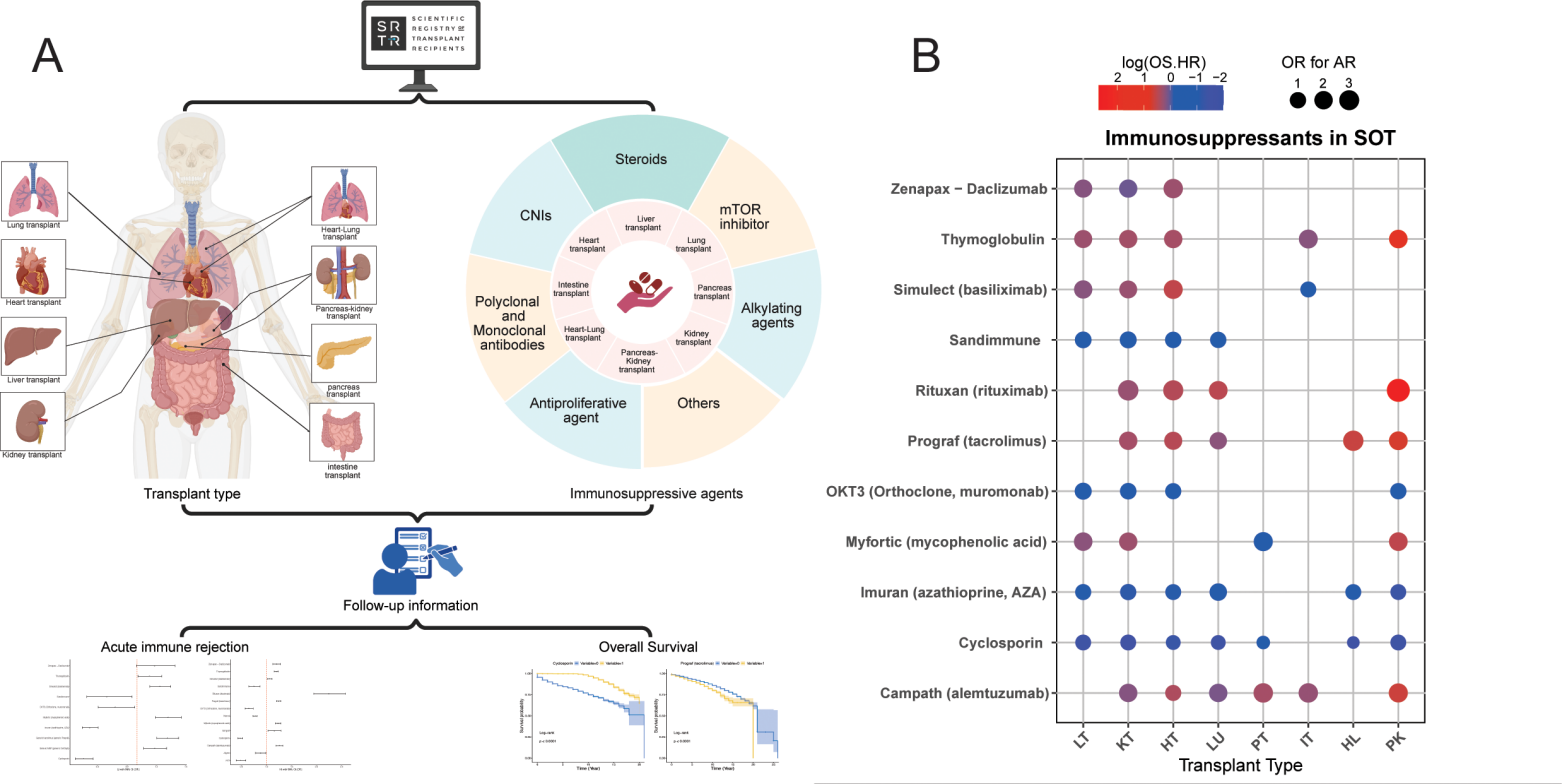
Study flowchart and main findings of this study. **(A)** Study Flowchart. Using the SRTR database to analyze how different immunosuppressants affect post transplantation acute rejection (AR) and overall survival in solid organ transplantation (SOT) patients. **(B)** The bubble plot illustrates the impact of different immunosuppressants on AR and overall survival (OS) in solid organ transplant (SOT) patients. To enhance clarity, hazard ratios were standardized by logarithmic transformation for visual clarity, with blue indicating survival-promoting effects (HR<1), red representing survival-compromising effects (HR>1), and bubble size reflecting odds ratios for AR.

This study analyzed diverse solid organ transplant (SOT) populations through the Scientific Registry of Transplant Recipients (SRTR) database to evaluate the immunosuppressants impacts on AR and overall survival (OS) across solid organ transplants(**Figure 1A**). The bubble plot revealed roles of common immunosuppressants in various organ transplant types(**Figure 1B**). The baseline characteristics of the study cohort, comprising 118,000 liver transplant (LT) recipients, 319,328 kidney transplant (KT) recipients, 46,157 heart transplant (HT) recipients, 25,789 lung transplant (LU) recipients, 6,361 pancreas transplant (PT) recipients, 2,175 intestine transplant (IT) recipients, 517 heart-lung transplant (HL) and 23101 pancreas-kidney transplant (PK), are presented in **Table 1**.

**Table 1:** Baseline characteristics of different transplant patients.

Transplant type	Characteristic		Non-AR group	AR group	P
LT	AR (NO. (%) )		113532 (96.21)	4468 (3.79)	
	Age (years,mean (SD) )		48.63 (17.41)	40.59 (21.48)	<0.001
	Sex (No. (%) )				<0.001
		F	40937 (36.10)	1920 (43.00)	
		M	72595 (63.90)	2548 (57.00)	
	Weight (kg,mean (SD) )		78.20 (27.02)	71.17 (31.38)	<0.001
	Height (cm, mean (SD) )		165.03 (26.61)	157.79 (34.00)	<0.001
	ABO (No. (%) )				0.02
		A	42576 (37.50)	1565 (35.00)	
		AB	5444 (4.80)	215 (4.80)	
		B	14934 (13.20)	621 (13.90)	
		O	50312 (44.30)	2056 (46.0)	
		Others	266 (0.20)	11 (0.20)	
	Follow-up status (No. (%) )				<0.001
		0	96678 (86.80)	2674 (66.50)	
		1	14665 (13.20)	1347 (33.50)	
KT	**Characteristic**		**Non-AR group**	**AR group**	**P**
	AR (NO. (%) )		304688 (95.42)	14640 (4.58)	
	Age (years,mean (SD) )		47.83 (15.62)	40.83 (17.17)	<0.001
	Sex (No. (%) )				<0.001
		F	120583 (39.60)	6104 (41.70)	
		M	184105 (60.40)	8536 (58.30)	
	Weight (kg,mean (SD) )		77.88 (21.63)	76.85 (23.74)	<0.001
	Height (cm, mean (SD) )		168.60 (15.31)	167.42 (16.98)	<0.001
	ABO (No. (%) )				<0.001
		A	110127 (36.10)	5062 (34.60)	
		AB	14042 (4.60)	604 (4.10)	
		B	39181 (12.90)	1926 (13.20)	
		O	137108 (45.00)	6855 (46.80)	
		Others	4230 (1.40)	193 (1.30)	
	Follow-up status (No. (%) )				<0.001
		0	279355 (92.20)	13120 (90.70)	
		1	23631 (7.80)	1343 (9.30)	
HT	**Characteristic**		**Non-AR group**	**AR group**	**P**
	AR (NO. (%) )		41246 (89.36)	4911 (10.64)	
	Age (years,mean (SD) )		46.48 (19.36)	39.75 (20.98)	<0.001
	Sex (No. (%) )				<0.001
		F	10877 (26.40)	1593 (32.40)	
		M	30369 (73.60)	3318 (67.60)	
	Weight (kg,mean (SD) )		73.51 (26.27)	71.21 (28.78)	<0.001
	Height (cm, mean (SD) )		165.07 (29.43)	161.86 (31.46)	<0.001
	ABO (No. (%) )				0.002
		A	17074 (41.40)	1909 (38.90)	
		AB	2119 (5.10)	279 (5.70)	
		B	5663 (13.70)	737 (15.00)	
		O	16217 (39.30)	1959 (39.90)	
		Others	173 (0.40)	27 (0.50)	
	Follow-up status (No. (%) )				<0.001
		0	36196 (88.30)	3120 (65.70)	
		1	4778 (11.70)	1629 (34.30)	
LU	**Characteristic**		**Non-AR group**	**AR group**	**P**
	AR (NO. (%) )		22049 (85.50)	3740 (14.50)	
	Age (years,mean (SD) )		52.45 (14.87)	50.65 (16.55)	<0.001
	Sex (No. (%) )				0.424
		F	9640 (43.70)	1662 (44.40)	
		M	12409 (56.30)	2078 (55.60)	
	Weight (kg,mean (SD) )		70.95 (18.75)	70.43 (18.80)	0.112
	Height (cm, mean (SD) )		168.48 (13.28)	168.53 (12.45)	0.838
	ABO (No. (%) )				0.236
		A	8806 (39.90)	1496 (40.00)	
		AB	839 (3.80)	149 (4.00)	
		B	2446 (11.10)	414 (11.10)	
		O	9868 (44.80)	1675 (44.80)	
		Others	90 (0.40)	6 (0.20)	
	Follow-up status (No. (%) )				<0.001
		0	15766 (72.60)	1678 (47.30)	
		1	5941 (27.40)	1873 (52.70)	
PT	**Characteristic**		**Non-AR group**	**AR group**	**P**
	AR (NO. (%) )		5831 (91.67)	530 (8.33)	
	Age (years,mean (SD) )		38.79 (13.96)	37.83 (12.21)	0.126
	Sex (No. (%) )				0.003
		F	2729 (46.80)	284 (53.60)	
		M	3102 (53.20)	246 (46.40)	
	Weight (kg,mean (SD) )		67.24 (22.36)	68.13 (18.91)	0.374
	Height (cm, mean (SD) )		162.08 (27.86)	164.51 (22.04)	0.05
	ABO (No. (%) )				0.341
		A	2383 (40.90)	209 (39.40)	
		AB	255 (4.40)	21 (4.00)	
		B	646 (11.10)	72 (13.60)	
		O	2497 (42.80)	226 (42.60)	
		Others	50 (0.90)	2 (0.40)	
	Follow-up status (No. (%) )				0.143
		0	5081 (88.60)	435 (86.30)	
		1	654 (11.40)	69 (13.70)	
IT	**Characteristic**		**Non-AR group**	**AR group**	**P**
	AR (NO. (%) )		1773 (81.52)	402 (18.48)	
	Age (years,mean (SD) )		22.38 (21.47)	22.64 (20.04)	0.824
	Sex (No. (%) )				0.954
		F	870 (49.10)	196 (48.80)	
		M	903 (50.90)	206 (51.20)	
	Weight (kg,mean (SD) )		39.53 (30.08)	41.95 (28.30)	0.141
	Height (cm, mean (SD) )		124.42 (48.28)	131.18 (45.87)	0.011
	ABO (No. (%) )				0.633
		A	654 (36.90)	151 (37.60)	
		AB	77 (4.30)	16 (4.00)	
		B	215 (12.10)	59 (14.70)	
		O	824 (46.50)	175 (43.50)	
		Others	3 (0.20)	1 (0.20)	
	Follow-up status (No. (%) )				<0.001
		0	1352 (77.30)	188 (51.10)	
		1	398 (22.70)	180 (48.90)	
HL	**Characteristic**		**Non-AR group**	**AR group**	**P**
	AR (NO. (%) )		440 (85.11)	77 (14.89)	
	Age (years,mean (SD) )		36.45 (14.86)	34.83 (14.54)	0.376
	Sex (No. (%) )				0.246
		F	263 (59.80)	40 (51.90)	
		M	177 (40.20)	37 (48.10)	
	Weight (kg,mean (SD) )		60.94 (18.30)	61.23 (19.84)	0.897
	Height (cm, mean (SD) )		161.75 (20.76)	163.58 (18.25)	0.468
	ABO (No. (%) )				0.843
		A	195 (44.30)	34 (44.20)	
		AB	23 (5.20)	6 (7.80)	
		B	50 (11.40)	7 (9.10)	
		O	169 (38.40)	29 (37.70)	
		Others	3 (0.70)	1 (1.30)	
	Follow-up status (No. (%) )				<0.001
		0	338 (77.50)	25 (33.30)	
		1	98 (22.50)	50 (66.70)	
PK	**Characteristic**		**Non-AR group**	**AR group**	**P**
	AR (NO. (%) )		22344 (96.72)	757 (3.28)	
	Age (years,mean (SD) )		40.39 (8.41)	37.53 (8.96)	<0.001
	Sex (No. (%) )				0.022
		F	8711 (39.00)	327 (43.20)	
		M	13632 (61.00)	430 (56.80)	
	Weight (kg,mean (SD) )		72.37 (14.95)	71.92 (15.03)	0.413
	Height (cm, mean (SD) )		170.66 (10.69)	170.04 (11.84)	0.116
	ABO (No. (%) )				<0.001
		A	6057 (27.10)	228 (30.10)	
		AB	667 (3.00)	25 (3.30)	
		B	1978 (8.90)	79 (10.40)	
		O	7876 (35.20)	308 (40.70)	
		Others	5766 (25.80)	117 (15.50)	
	Follow-up status (No. (%) )				<0.001
		0	20689 (93.20)	639 (86.90)	
		1	1505 (6.80)	96 (13.10)	

P-value less than 0.05 means statistically significant.

LT, liver transplant; KT, kidney transplant; HT, heart transplant; LU, lung transplant; PT, pancreas transplant; IT, intestinal transplant; HL, heart-lung transplant; KP,kidney-pancreas transplant; F, female; M, male; ABO, blood type; AR, acute rejection episodes during the follow-up period.

We analyzed rejection rates across different organ transplants, recipients were stratified into two groups: nonacute rejection (non-AR) and AR. The incidence of AR across transplant types was as follows: LT - 3.79% (n=4,468), KT - 4.58% (n=14,640), HT - 10.64% (n=4,911), LU - 14.50% (n=3,740), PT - 8.33% (n=530), IT - 18.48% (n=402), HL-14.89%(n=77) and PK-3.28%(n=757), as detailed in **Table 1**.

**Figure 2:**
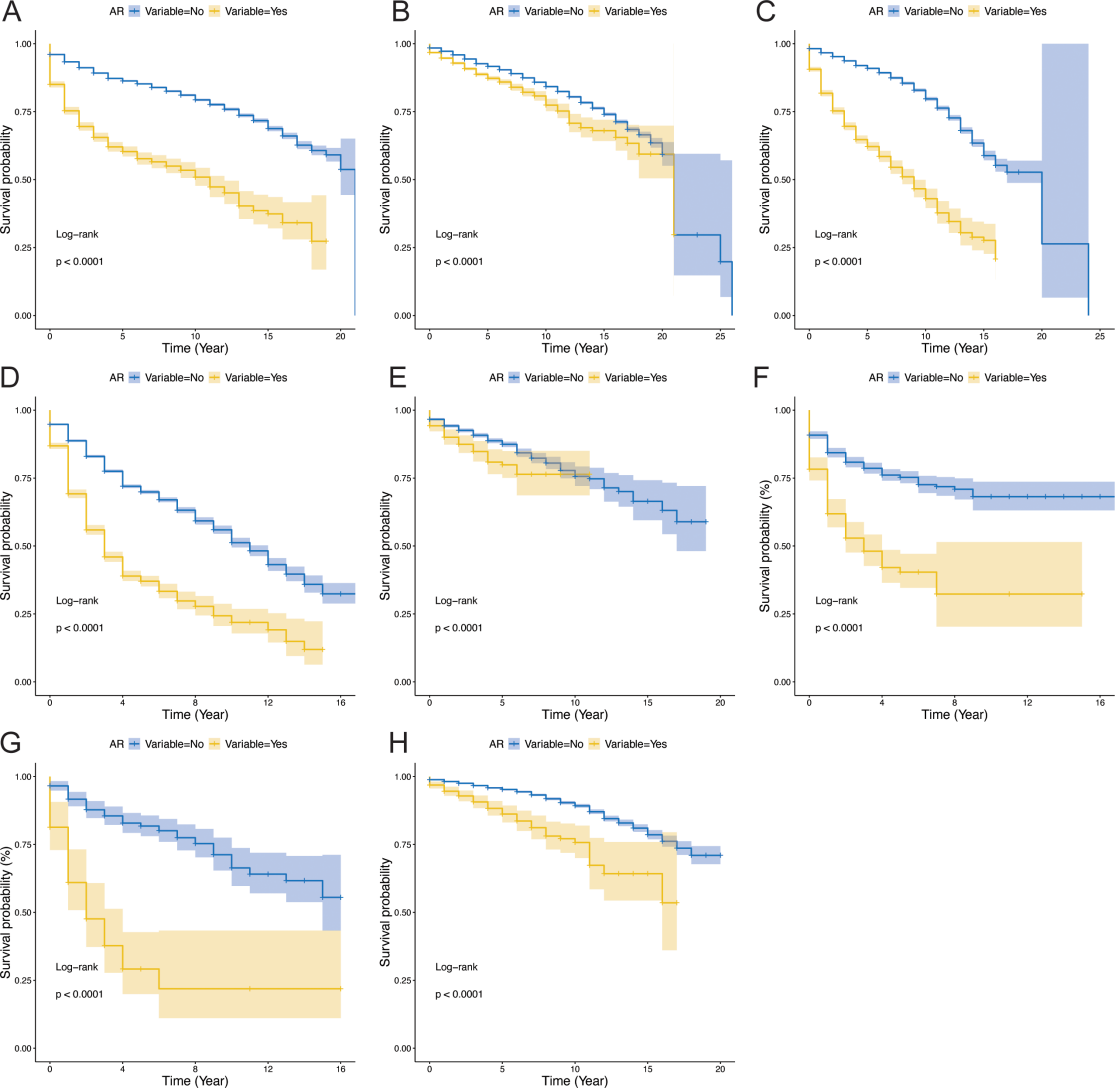
Impact of acute rejection on survival in transplant patients (P-value < 0.05 was considered statistically significant). **(A)** Impact of AR on survival in liver transplant; **(B)** Impact of AR on survival in kidney transplant patients; **(C)** Impact of AR on survival in patients heart transplant patients; **(D)** Impact of AR on survival in lung transplant patients; **(E)** Impact of AR on survival in pancreas transplant patients; **(F)** Impact of AR on survival in intestinal transplant patients; **(G)** Impact of AR on survival in heart-lung transplant patients; **(H)** Impact of AR on survival in pancreas-kidney transplant patients.

Kaplan–Meier survival analysis revealed significantly lower overall survival (OS) in recipients who experienced AR than in their rejection-free counterparts (**Figure 2**, P < 0.001). Notably, the relatively limited sample size of IT and HL recipients may constrain the statistical power of related analyses. Furthermore, baseline characteristics, including age, sex, body mass index (BMI) and ABO blood type, exhibited differential distributions between rejection groups (**Table 1**); these parameters may confound rejection incidence and clinical outcomes, and further investigation into their synergistic effects in future mechanistic studies is warranted.

### 3.2 Liver transplant

#### 3.2.1 Impact of immunosuppressants on patient survival

**Figure 3:**
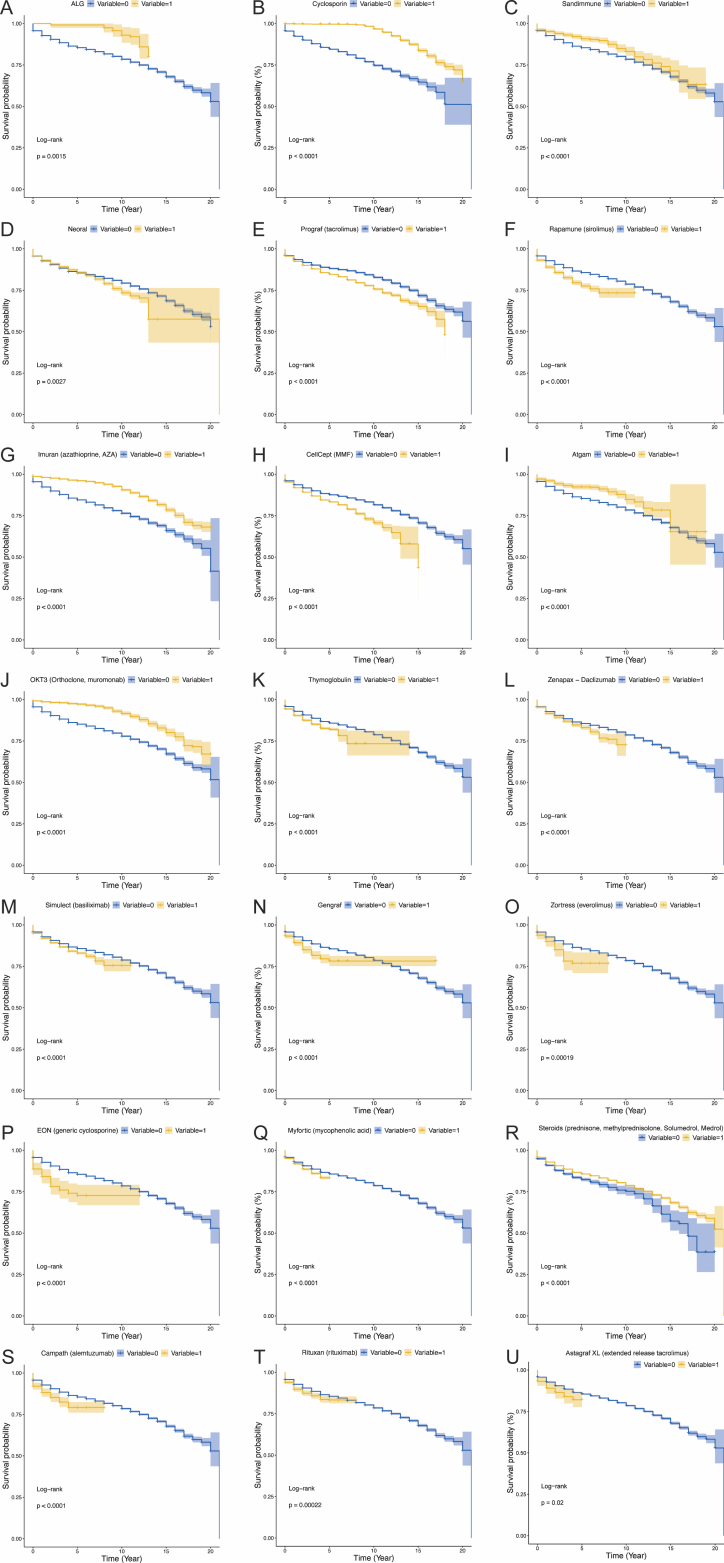
The influence of various immunosuppressants on survival in liver transplant patients. *P*-value less than 0.05 was considered significant. **(A)** The influence of ALG on survival in liver transplant patients. **(B)** The influence of Cyclosporin on survival in liver transplant patients. **(C)** The influence of Sandimmune on survival in liver transplant patients. **(D)** The influence of Neoral on survival in liver transplant patients. **(E)** The influence of Prograf (tacrolimus) on survival in liver transplant patients. **(F)** The influence of Rapamune (sirolimus) on survival in liver transplant patients. **(G)** The influence of Imuran (azathioprine, AZA) on survival in liver transplant patients. **(H)** The influence of CellCept (MMF) on survival in liver transplant patients. **(I)** The influence of Atgam on survival in liver transplant patients. **(J)** The influence of OKT3 (Orthoclone, muromonab) on survival in liver transplant patients. **(K)** The influence of Thymoglobulin on survival in liver transplant patients. **(L)** The influence of Zenapax−Daclizumab on survival in liver transplant patients. **(M)** The influence of Simulect (basiliximab) on survival in liver transplant patients. **(N)** The influence of Gengraf on survival in liver transplant patients. **(O)** The influence of Zortress (everolimus) on survival in liver transplant patients. **(P)** The influence of EON (generic cyclosporine) on survival in liver transplant patients. **(Q)** The influence of Myfortic (mycophenolic acid) on survival in liver transplant patients. **(R)** The influence of Steroids on survival in liver transplant patients. **(S)** The influence of Campath (alemtuzumab) on survival in liver transplant patients. **(T)** The influence of Rituxan (rituximab) on survival in liver transplant patients. **(U)** The influence of Astagraf XL (extended release tacrolimus) on survival in liver transplant patients.

In LT, patients receiving anti-lymphocyte globulin (ALG), cyclosporine (Cyclosporin), Sandimmune, AZA (Imuran), ATG(Atgam), OKT3 monoclonal antibody and Steroids had significantly better survival than those who did not receive these immunosuppressants, possibly related to the synergistic effect of classic immunosuppressive regimens (such as polyclonal antibodies combined with antimetabolite drugs) and more controllable immunosuppression intensity (**Figures 3A–C, G, I, J, R**; all P<0.05). However, patients receiving Neoral, tacrolimus (Prograf), sirolimus (Rapamune), MMF (CellCep), Thymoglobulin, daclizumab (Zenapax), basiliximab (Simulect), everolimus (Zortress), generic cyclosporine (EON), mycophenolic acid (Myfortic), alemtuzumab (Campath), rituximab (Rituxan), and extended-release tacrolimus (Astagraf XL) had significantly lower survival rates, suggesting that potent lymphocyte-depleting drugs (such as Campath) and high-dose calcineurin inhibitors (CNIs; such as Astagraf XL) may increase the risk of opportunistic infections, metabolic disorders, and chronic graft dysfunction due to excessive immunosuppression, thereby impairing long-term survival outcomes (**Figures 3D–F, H, K–M, O–Q, S–U**; all P<0.05). It’s interesting to note that while gengraf increases long-term survival, it lowers short-term survival rates in liver transplant recipients (**Figure 3N**, P<0.05).

#### 3.2.2 Impact of immunosuppressants on AR

In LT, generic tacrolimus (OR: 1.31; 95% CI: 1.21–1.42; P<0.001), mycophenolic acid (Myfortic) (OR: 1.32; 95% CI: 1.19–1.45; P<0.001), basiliximab (Simulect) (OR: 1.23; 95% CI: 1.13–1.34; P<0.001), generic MMF (generic CellCept) (OR: 1.18; 95% CI: 1.07–1.30; P<0.001), thymoglobulin (OR: 1.13; 95% CI: 1.01–1.25; P<0.001), and Zenapax-Daclizumab (OR: 1.18; 95% CI: 1.00–1.39; P<0.001) were associated with an increased risk of Acute Rejection. Conversely, the use of AZA (Imuran) (OR: 0.52; 95% CI: 0.44–0.60; P<0.001), Cyclosporin (OR: 0.46; 95% CI: 0.37–0.55; P<0.001), Sandimmune (OR: 0.69; 95% CI: 0.49–0.95; P<0.001), or OKT3 (Orthoclone, muromonab) (OR: 0.78; 95% CI: 0.61–0.98; P<0.001) appeared to reduce the risk of AR (**Table 2**; **Supplementary Figure S5**).

**Table 2:** Impact of immunosuppressants on AR in different organ transplant patients.

Transplant type	Drug name	OR (95% CI)	P
LT	Imuran (azathioprine, AZA)	0.52	1.50E-16
		(0.44-0.60)	
	Cyclosporin	0.46	5.08E-15
		(0.37-0.55)	
	Generic tacrolimus (generic Prograf)	1.31	2.07E-10
		(1.21-1.42)	
	Myfortic (mycophenolic acid)	1.32	6.84E-08
		(1.19-1.45)	
	Simulect (basiliximab)	1.23	1.28E-06
		(1.13-1.34)	
	Generic MMF (generic CellCept)	1.18	9.10E-04
		(1.07-1.30)	
	Thymoglobulin	1.13	2.57E-02
		(1.01-1.25)	
	Sandimmune	0.69	2.90E-02
		(0.49-0.95)	
	OKT3 (Orthoclone, muromonab)	0.78	3.79E-02
		(0.61-0.98)	
	Zenapax - Daclizumab	1.18	4.98E-02
		(1.00-1.39)	
KT	**Drug name**	**OR (95% CI)**	**P**
	Cyclosporin	0.47	2.21E-46
		(0.43-0.52)	
	Imuran (azathioprine, AZA)	0.56	4.50E-46
		(0.52-0.61)	
	Prograf (tacrolimus)	1.24	7.75E-33
		(1.20-1.28)	
	Rituxan (rituximab)	2.24	2.22E-29
		(1.94-2.57)	
	Thymoglobulin	1.19	1.19E-24
		(1.15-1.23)	
	Myfortic (mycophenolic acid)	1.23	5.41E-22
		(1.18-1.28)	
	Neoral	0.77	5.09E-21
		(0.73-0.81)	
	Campath (alemtuzumab)	1.25	1.53E-17
		(1.19-1.32)	
	ALG	0.48	8.01E-13
		(0.39-0.58)	
	OKT3 (Orthoclone, muromonab)	0.64	1.30E-11
		(0.57-0.73)	
	Zenapax - Daclizumab	1.20	2.18E-08
		(1.13-1.28)	
	Sandimmune	0.75	1.39E-04
		(0.64-0.87)	
	Simulect (basiliximab)	1.06	6.29E-03
		(1.02-1.10)	
	Gengraf	1.15	1.17E-02
		(1.03-1.28)	
	Atgam	0.88	3.49E-02
		(0.78-0.99)	
	Steroids (prednisone, methylprednisolone, Solumedrol, Medrol)	0.92	5.21E-02
		(0.85-1.00)	
	Generic cyclosporine	1.67	5.61E-02
		(0.94-2.73)	
HT	**Drug name**	**OR (95% CI)**	**P**
	Imuran (azathioprine, AZA)	0.48	3.90E-58
		(0.44-0.53)	
	Cyclosporin	0.27	6.53E-48
		(0.22-0.32)	
	Simulect (basiliximab)	1.52	3.85E-28
		(1.41-1.63)	
	CellCept (MMF)	1.40	6.00E-25
		(1.31-1.49)	
	Zenapax - Daclizumab	1.72	2.18E-24
		(1.55-1.91)	
	OKT3 (Orthoclone, muromonab)	0.54	1.03E-12
		(0.45-0.63)	
	Generic tacrolimus (generic Prograf)	1.31	7.16E-11
		(1.21-1.42)	
	Gengraf	1.50	9.27E-10
		(1.31-1.7)	
	Thymoglobulin	1.26	3.89E-09
		(1.17-1.36)	
	Sandimmune	0.61	2.53E-08
		(0.51-0.72)	
	Rituxan (rituximab)	2.07	1.41E-06
		(1.53-2.77)	
	Prograf (tacrolimus)	1.14	2.49E-05
		(1.07-1.21)	
	Generic cyclosporine	2.05	7.34E-05
		(1.42-2.9)	
	Generic MMF (generic CellCept)	1.19	1.03E-04
		(1.09-1.3)	
	Myfortic (mycophenolic acid)	1.32	3.24E-04
		(1.13-1.53)	
	Campath (alemtuzumab)	0.51	1.01E-03
		(0.34-0.75)	
	EON (generic cyclosporine)	1.86	1.02E-02
		(1.13-2.92)	
	NRATG /NRATS	0.42	1.72E-02
		(0.19-0.8)	
	Neoral	0.92	2.28E-02
		(0.86-0.99)	
	Rapamune (sirolimus)	1.22	4.67E-02
		(1.00-1.47)	
LU	**Drug name**	**OR (95% CI)**	**P**
	Cyclosporin	0.27	3.58E-09
		(0.17-0.4)	
	Atgam	0.61	8.25E-08
		(0.51-0.73)	
	Generic tacrolimus (generic Prograf)	1.27	9.10E-08
		(1.16-1.38)	
	Sandimmune	0.58	1.81E-05
		(0.45-0.74)	
	Prograf (tacrolimus)	0.86	4.98E-05
		(0.8-0.93)	
	Zenapax - Daclizumab	0.74	1.16E-04
		(0.63-0.86)	
	Neoral	1.19	8.36E-04
		(1.07-1.32)	
	Generic MMF (generic CellCept)	1.17	1.14E-03
		(1.06-1.29)	
	Campath (alemtuzumab)	1.25	2.47E-03
		(1.08-1.43)	
	Myfortic (mycophenolic acid)	0.76	5.08E-03
		(0.62-0.92)	
	EON (generic cyclosporine)	2.30	8.37E-03
		(1.20-4.17)	
	Rituxan (rituximab)	1.38	3.07E-02
		(1.02-1.84)	
	Imuran (azathioprine, AZA)	0.93	5.03E-02
		(0.86-1.00)	
PT	**Drug name**	**OR (95% CI)**	**P**
	Campath (alemtuzumab)	1.74	2.54E-06
		(1.38-2.19)	
	Myfortic (mycophenolic acid)	1.59	4.72E-04
		(1.22-2.06)	
	Steroids (prednisone, methylprednisolone, Solumedrol, Medrol)	2.47	7.17E-04
		(1.52-4.35)	
	Imuran (azathioprine, AZA)	0.35	3.56E-03
		(0.16-0.66)	
	Generic tacrolimus (generic Prograf)	1.64	6.52E-03
		(1.13-2.32)	
	Zenapax - Daclizumab	0.63	7.94E-03
		(0.44-0.87)	
	Cyclosporin	0.13	4.07E-02
		(0.01-0.58)	
	Thymoglobulin	1.20	4.36E-02
		(1.01-1.44)	
IT	**Drug name**	**OR (95% CI)**	**P**
	OKT3 (Orthoclone, muromonab)	2.17	2.31E-04
		(1.42-3.25)	
	Campath (alemtuzumab)	1.71	2.92E-04
		(1.27-2.27)	
	Thymoglobulin	1.48	5.52E-04
		(1.18-1.84)	
	Simulect (basiliximab)	0.49	5.61E-03
		(0.29-0.79)	
	Atgam	2.24	3.97E-02
		(1.00-4.71)	
	CellCept (MMF)	0.77	5.31E-02
		(0.59-1.00)	
HL	**Drug name**	**OR (95% CI)**	**P**
	Prograf (tacrolimus)	2.20	3.45E-03
		(1.31-3.8)	
	Imuran (azathioprine, AZA)	0.48	1.04E-02
		(0.27-0.83)	
	CellCept (MMF)	1.83	1.78E-02
		(1.12-3.05)	
	Cyclosporin	0.10	2.13E-02
		(0.01-0.45)	
PK	**Drug name**	**OR (95% CI)**	**P**
	Imuran (azathioprine, AZA)	0.46	1.21E-08
		(0.34-0.59)	
	Cyclosporin	0.47	5.15E-06
		(0.34-0.64)	
	Rituxan (rituximab)	3.95	2.08E-05
		(1.98-7.13)	
	Prograf (tacrolimus)	1.43	3.74E-05
		(1.21-1.7)	
	Thymoglobulin	1.35	6.10E-05
		(1.16-1.56)	
	OKT3 (Orthoclone, muromonab)	0.58	8.32E-05
		(0.43-0.75)	
	Steroids (prednisone, methylprednisolone, Solumedrol, Medrol)	2.01	8.34E-05
		(1.44-2.89)	
	Campath (alemtuzumab)	1.57	2.38E-04
		(1.23-1.99)	
	Myfortic (mycophenolic acid)	1.37	1.46E-03
		(1.12-1.66)	
	Neoral	0.64	2.37E-03
		(0.48-0.85)	
	Generic tacrolimus (generic Prograf)	1.38	1.81E-02
		(1.05-1.78)	
	Sandimmune	0.35	2.09E-02
		(0.13-0.77)	
	Generic MMF (generic CellCept)	1.39	4.47E-02
		(0.99-1.88)	

P-value less than 0.05 means statistically significant.

OR, odds ratio.

### 3.3 Kidney transplant

#### 3.3.1 Impact of immunosuppressants on patient survival

**Figure 4:**
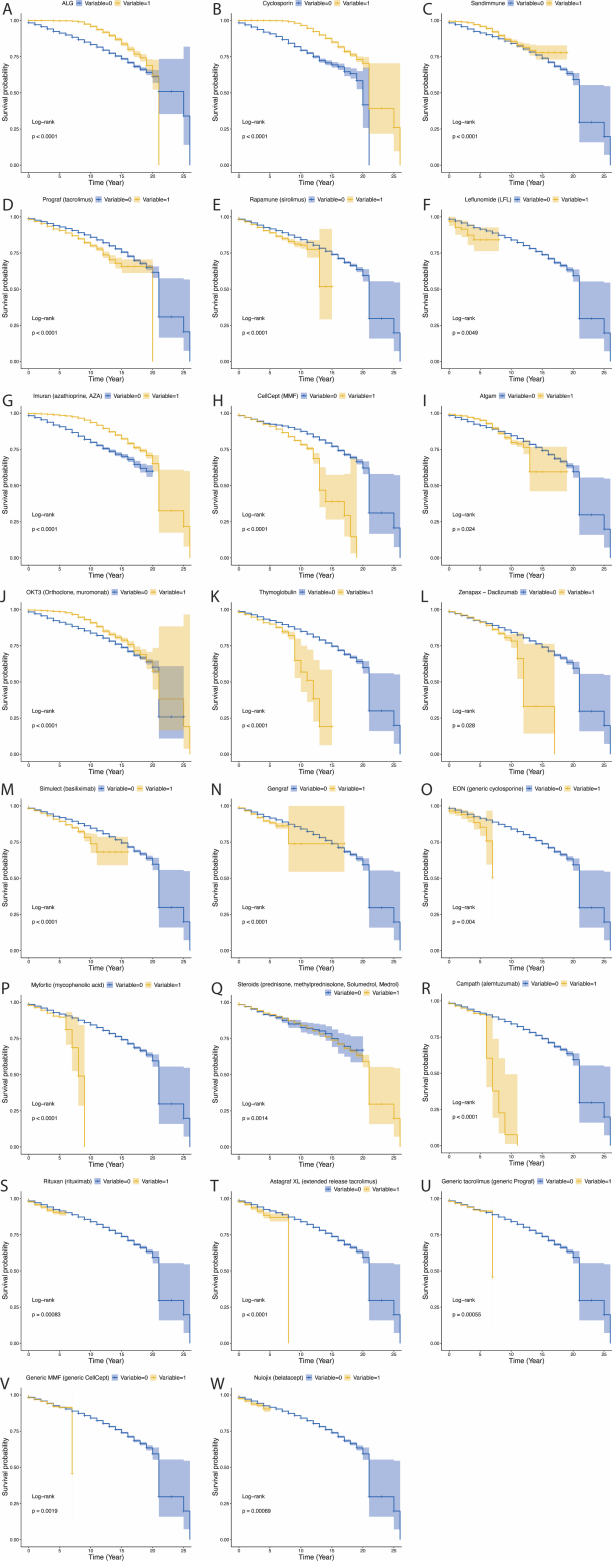
The influence of various immunosuppressants on survival in kidney transplant patients. *P*-value less than 0.05 was considered significant. **(A)** The influence of ALG on survival in kidney transplant patients. **(B)** The influence of Cyclosporin on survival in kidney transplant patients. **(C)** The influence of Sandimmune on survival in kidney transplant patients. **(D)** The influence of Prograf (tacrolimus) on survival in kidney transplant patients. **(E)** The influence of Rapamune (sirolimus) on survival in kidney transplant patients. **(F)** The influence of Leflunomide (LFL) on survival in kidney transplant patients. **(G)** The influence of Imuran (azathioprine, AZA) on survival in kidney transplant patients. **(H)** The influence of CellCept (MMF) on survival in kidney transplant patients. **(I)** The influence of Atgam on survival in kidney transplant patients. **(J)** The influence of OKT3 (Orthoclone, muromonab) on survival in kidney transplant patients. **(K)** The influence of Thymoglobulin on survival in kidney transplant patients. **(L)** The influence of Zenapax−Daclizumab on survival in kidney transplant patients. **(M)** The influence of Simulect (basiliximab) on survival in kidney transplant patients. **(N)** The influence of Gengraf on survival in kidney transplant patients. **(O)** The influence of EON (generic cyclosporine) on survival in kidney transplant patients. **(P)** The influence of Myfortic (mycophenolic acid) on survival in kidney transplant patients. **(Q)** The influence of Steroids on survival in kidney transplant patients. **(R)** The influence of Campath (alemtuzumab) on survival in kidney transplant patients. **(S)** The influence of Rituxan (rituximab) on survival in kidney transplant patients. **(T)** The influence of Astagraf XL on survival in kidney transplant patients. **(U)** The influence of Generic tacrolimus (generic Prograf) on survival in kidney transplant patients. **(V)** The influence of Generic MMF (generic CellCept) on survival in kidney transplant patients. **(W)** The influence of Nulojix (belatacept) on survival in kidney transplant patients.

In KT, patients receiving ALG, cyclosporine (Cyclosporin), Sandimmune, AZA (Imuran), ATG(Atgam) and OKT3 monoclonal antibody, had significantly better survival than those who did not receive these drugs (**Figures 4A–C, G, I, J**; all P<0.05). However, patients receiving tacrolimus (Prograf), sirolimus (Rapamune), Leflunomide (LFL), MMF (CellCept), Thymoglobulin, Zenapax-Daclizumab, basiliximab (Simulect), gengraf, generic cyclosporine (EON), and mycophenolic acid (Myfortic) had significantly lower survival (**Figures 4D–F, H, K–P**; all P<0.05). Although p is less than 0.05, some drugs show no significant effect in improving patient survival rates (**Figures 4Q–W**).

#### 3.3.2 Impact of immunosuppressants on AR

In KT, tacrolimus (Prograf) (OR: 1.24; 95% CI: 1.2–1.28; P<0.001), rituximab (Rituxan) (OR: 2.24; 95% CI: 1.94–2.57; P<0.001), thymoglobulin (OR: 1.19; 95% CI: 1.15–1.23; P<0.001), mycophenolic acid (Myfortic) (OR: 1.23; 95% CI: 1.18–1.28; P<0.001), alemtuzumab (Campath) (OR: 1.25; 95% CI: 1.19–1.32; P<0.001), Zenapax-Daclizumab (OR: 1.20; 95% CI: 1.13–1.28; P<0.001), basiliximab (Simulect) (OR: 1.06; 95% CI: 1.02–1.1; P<0.001), gengraf (OR: 1.15; 95% CI: 1.03–1.28; P<0.001) and generic cyclosporine (OR: 1.67; 95% CI: 0.94–2.73; P<0.001) were associated with an increased risk of AR. Cyclosporin (OR: 0.47; 95% CI: 0.43–0.52; P<0.001), AZA (Imuran) (OR: 0.56; 95% CI: 0.52–0.61; P<0.001), Neoral (OR: 0.77; 95% CI: 0.73–0.81; P<0.001), ALG (OR: 0.48; 95% CI: 0.39–0.58; P<0.001), OKT3 (OR: 0.64; 95% CI: 0.57–0.73; P<0.001), Sandimmune (OR: 0.75; 95% CI: 0.64–0.87; P<0.001), Atgam (OR: 0.88; 95% CI: 0.78–0.99; P<0.001), and steroids (OR: 0.92; 95% CI: 0.85–1.00; P<0.001) were associated with a reduced risk of AR (**Table 2**; **Supplementary Figure S5**).

### 3.4 Heart transplant

#### 3.4.1 Impact of immunosuppressants on patient survival

**Figure 5:**
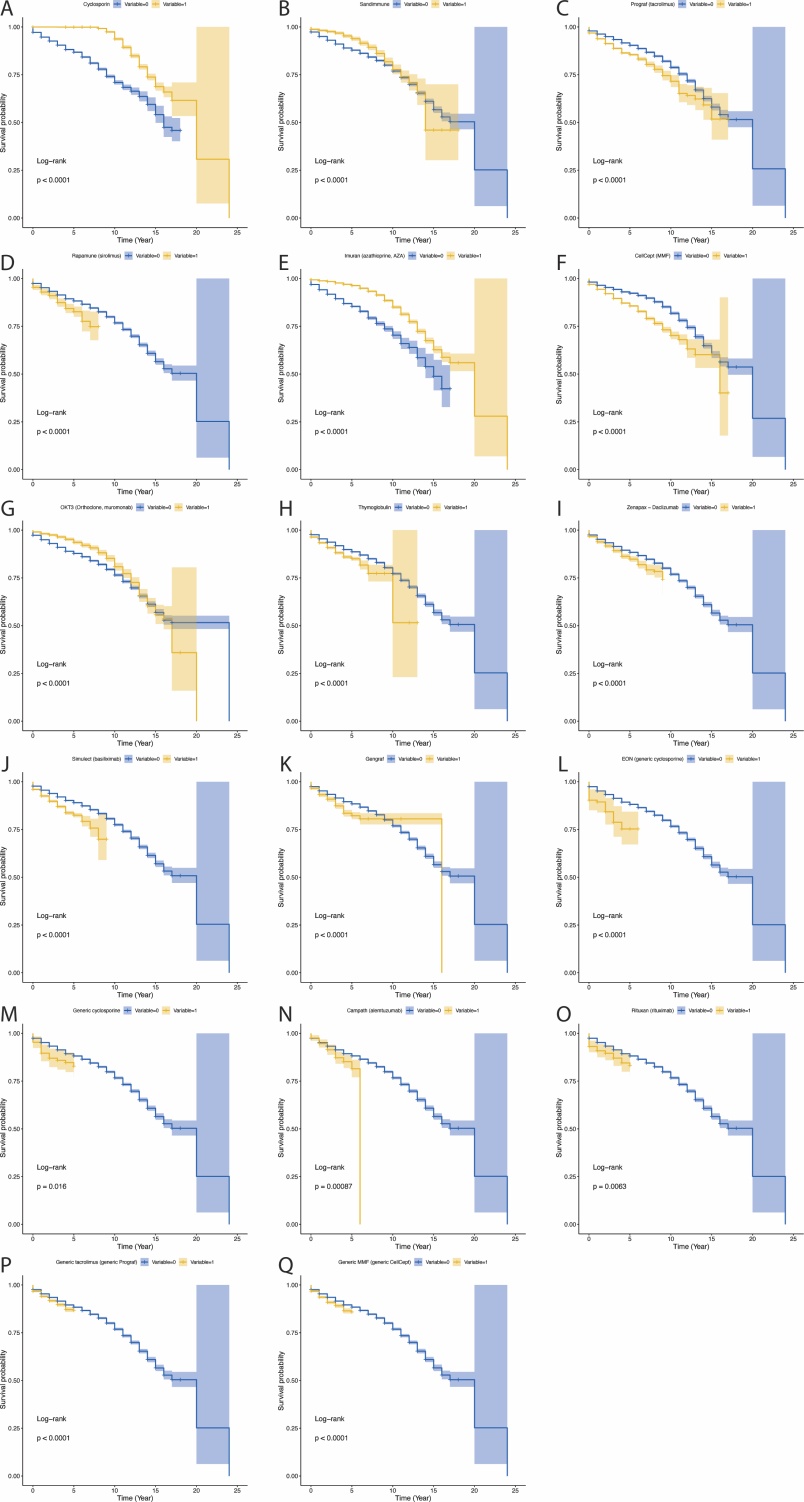
The influence of various immunosuppressants on survival in heart transplant patients. *P*-value less than 0.05 was considered significant. **(A)** The influence of Cyclosporin on survival in heart transplant patients. **(B)** The influence of Sandimmune on survival in heart transplant patients. **(C)** The influence of Prograf (tacrolimus) on survival in heart transplant patients. **(D)** The influence of Rapamune (sirolimus) on survival in heart transplant patients. **(E)** The influence of Imuran (azathioprine, AZA) on survival in heart transplant patients. **(F)** The influence of CellCept (MMF) on survival in heart transplant patients. **(G)** The influence of OKT3 (Orthoclone, muromonab) on survival in heart transplant patients. **(H)** The influence of Thymoglobulin on survival in heart transplant patients. **(I)** The influence of Zenapax−Daclizumab on survival in heart transplant patients. **(J)** The influence of Simulect (basiliximab) on survival in heart transplant patients. **(K)** The influence of Gengraf on survival in heart transplant patients. **(L)** The influence of EON (generic cyclosporine) on survival in heart transplant patients. **(M)** The influence of Generic cyclosporine on survival in heart transplant patients. **(N)** The influence of Campath (alemtuzumab) on survival in heart transplant patients. **(O)** The influence of Rituxan (rituximab) on survival in heart transplant patients. **(P)** The influence of Generic tacrolimus (generic Prograf) on survival in heart transplant patients. **(Q)** The influence of Generic MMF (generic CellCept) on survival in heart transplant patients.

In HT, patients receiving Cyclosporin, Sandimmune, AZA (Imuran) and OKT3 had significantly better post transplantation survival than those who did not receive these drugs, the mechanism of which may be related to the synergistic effect of classic immunosuppressive regimens (polyclonal antibodies combined with antimetabolite drugs) (**Figures 5A, B, E, G**; all P<0.05). Conversely, patients receiving tacrolimus (Prograf) sirolimus (Rapamune), MMF (CellCept), anti-human T-cell globulin (Thymoglobulin), daclizumab (Zenapax), basiliximab (Simulect), generic cyclosporine (EON), generic cyclosporine, alemtuzumab (Campath), rituximab (Rituxan), Generic tacrolimus and generic MMF had significantly lower survival rates, suggesting that potent lymphocyte-depleting drugs (such as Campath) and high-dose CNIs (such as Astagraf XL) may lead to excessive immunosuppression, increasing the risk of infection and metabolic complications (**Figures 5C, D, F, H–J, L–Q**; all P<0.05). Although p is less than 0.05, some drugs show no significant effect in improving patient survival rates (**Figure 5K**).

#### 3.4.2 Impact of immunosuppressants on AR

In HT, patients receiving Simulect (basiliximab) (OR: 1.52; 95% CI: 1.41–1.63; P<0.001), MMF (CellCept) (OR: 1.40; 95% CI: 1.31–1.49; P<0.001), Zenapax–Daclizumab (OR: 1.72; 95% CI: 1.55–1.91; P<0.001), generic tacrolimus (generic Prograf) (OR: 1.31; 95% CI: 1.21–1.42; P<0.001), gengraf (OR: 1.50; 95% CI: 1.31–1.70; P<0.001), thymoglobulin (OR: 1.26; 95% CI: 1.17–1.36; P<0.001), rituximab (Rituxan) (OR: 2.07; 95% CI: 1.53–2.77; P<0.001), tacrolimus (Prograf) (OR: 1.14; 95% CI: 1.07–1.21; P<0.001), generic cyclosporine (OR: 2.05; 95% CI: 1.42–2.90; P<0.001), generic MMF (generic CellCept) (OR: 1.19; 95% CI: 1.09–1.30; P<0.001), mycophenolic acid (Myfortic) (OR: 1.32; 95% CI: 1.13–1.53; P<0.001), generic cyclosporine (EON) (OR: 1.86; 95% CI: 1.13–2.92; P<0.001), and sirolimus (Rapamune) (OR: 1.22; 95% CI: 1.00–1.47; P<0.001) had a higher risk of AR. Conversely, patients receiving AZA (Imuran) (OR: 0.48; 95% CI: 0.44–0.53; P<0.001), Cyclosporin (OR: 0.27; 95% CI: 0.22–0.32; P<0.001), OKT3 (Orthoclone, muromonab) (OR: 0.54; 95% CI: 0.45–0.63; P<0.001), Sandimmune (OR: 0.61; 95% CI: 0.51–0.72; P<0.001), alemtuzumab (Campath) (OR: 0.51; 95% CI: 0.34–0.75; P<0.001), NRATG/NRATS (OR: 0.42; 95% CI: 0.19–0.80; P<0.001), and Neoral (OR: 0.92; 95% CI: 0.86–0.99; P<0.001) had a lower risk of AR (**Table 2**; **Supplementary Figure S5**).

### 3.5 Lung transplant

#### 3.5.1 Impact of immunosuppressants on patient survival

**Figure 6:**
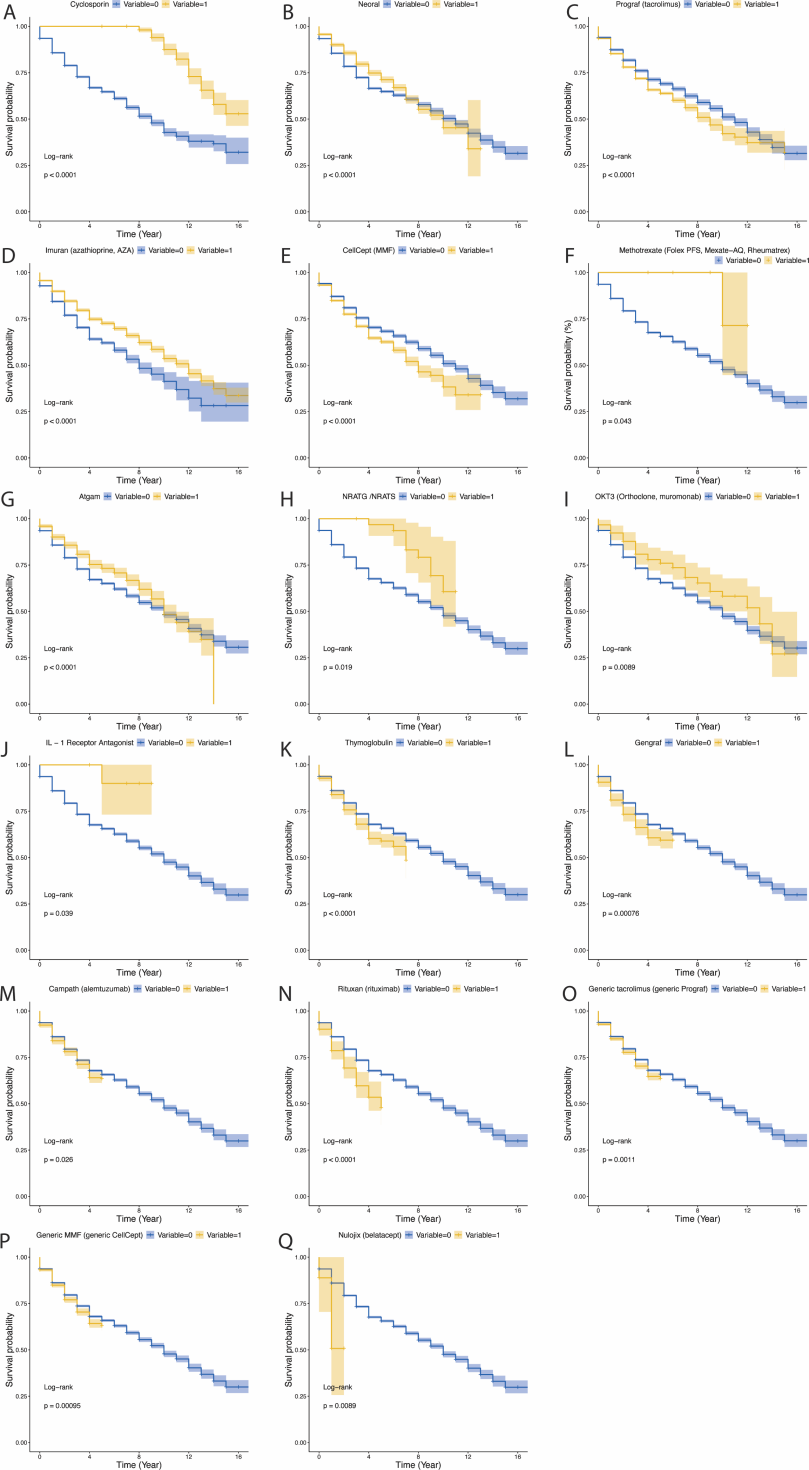
The influence of various immunosuppressants on survival in lung transplant patients. *P*-value less than 0.05 was considered significant. **(A)** The influence of Cyclosporin on survival in lung transplant patients. **(B)** The influence of Neoral on survival in lung transplant patients. **(C)** The influence of Prograf (tacrolimus) on survival in lung transplant patients. **(D)** The influence of Imuran (azathioprine, AZA) on survival in lung transplant patients. **(E)** The influence of CellCept (MMF) on survival in lung transplant patients. **(F)** The influence of Methotrexate (Folex PFS, Mexate−AQ, Rheumatrex) on survival in lung transplant patients. **(G)** The influence of Atgam on survival in lung transplant patients. **(H)** The influence of NRATG/NRATS on survival in lung transplant patients. **(I)** The influence of OKT3 (Orthoclone, muromonab) on survival in lung transplant patients. **(J)** The influence of IL−1 Receptor Antagonist on survival in lung transplant patients. **(K)** The influence of Thymoglobulin on survival in lung transplant patients. **(L)** The influence of Gengraf on survival in lung transplant patients. **(M)** The influence of Campath (alemtuzumab) on survival in lung transplant patients. **(N)** The influence of Rituxan (rituximab) on survival in lung transplant patients. **(O)** The influence of Generic tacrolimus (generic Prograf) on survival in lung transplant patients. **(P)** The influence of Generic MMF (generic CellCept) on survival in lung transplant patients. **(Q)** The influence of Nulojix (belatacept) on survival in lung transplant patients.

In LU, patients receiving cyclosporine (Cyclosporin), Neoral, AZA (Imuran), Methotrexate, ATG (Atgam), NRATG/NRATS, OKT3 and IL-1 Receptor Antagonist showed a significant survival advantage(**Figures 6A, B, D, F–J**; all P<0.05). However, tacrolimus (Prograf), MMF (CellCept), thymoglobulin, gengraf, alemtuzumab (Campath), rituximab (Rituxan), generic tacrolimus, generic MMF (generic CellCept) and belatacept (Nulojix) were associated with significantly decreased survival rates (**Figures 6C, E, K–Q**; all P<0.05).

#### 3.5.2 Impact of immunosuppressants on AR

In LU, the use of certain immunosuppressants was associated with an increased risk of AR, whereas other drugs were associated with a lower risk. Generic tacrolimus (generic Prograf) (OR: 1.27; 95% CI: 1.16–1.38; P<0.001), Neoral (OR: 1.19; 95% CI: 1.07–1.32; P<0.001), generic MMF (generic CellCept) (OR: 1.17; 95% CI: 1.06–1.29; P<0.001), alemtuzumab (Campath) (OR: 1.25; 95% CI: 1.08–1.43; P<0.001), EON (generic cyclosporine) (OR: 2.30; 95% CI: 1.20–4.17; P<0.001), and rituximab (OR: 1.38; 95% CI: (1.02–1.84); P<0.001) were associated with an increased risk of AR. Conversely, Cyclosporin (OR: 0.27; 95% CI: 0.17–0.40; P<0.001), Atgam (OR: 0.61; 95% CI: 0.51–0.73; P<0.001), Sandimmune (OR: 0.58; 95% CI: 0.45–0.74; P<0.001), tacrolimus (Prograf) (OR: 0.86; 95% CI: 0.80–0.93; P<0.001), Zenapax-Daclizumab (OR: 0.74; 95% CI: 0.63–0.86; P<0.001), mycophenolic acid (Myfortic) (OR: 0.76; 95% CI: 0.62–0.92; P<0.001), and AZA (Imuran) (OR: 0.93; 95% CI: 0.86–1.00; P<0.001) were associated with a reduced risk of AR (**Table 2**; **Supplementary Figure S5**).

### 3.6 Pancreas transplant

#### 3.6.1 Impact of immunosuppressants on patient survival

In PT, patients receiving cyclosporine (Cyclosporin), Neoral, MMF (CellCept), antithymocyte globulin (Atgam), and mycophenolic acid (Myfortic) had significantly increased post transplantation survival rates compared with those of the untreated group (**Supplementary Figures S1A–E**; all P<0.05). However, steroids, alemtuzumab (Campath) and rituximab (Rituxan) were associated with significantly decreased survival rates (**Supplementary Figures S1F–H**; all P<0.05).

#### 3.6.2 Impact of immunosuppressants on AR

In PT, Campath (OR: 1.74; 95% CI: 1.38–2.19; P<0.001), Myfortic (OR: 1.59; 95% CI: 1.22–2.06; P<0.001), Steroids (OR: 2.47; 95% CI: 1.52–4.35; P<0.001), and generic tacrolimus (OR: 1.64; 95% CI: 1.13–2.32; P<0.001), thymoglobulin (OR: 1.20; 95% CI: 1.01–1.44; P<0.001) were associated with an increased risk of AR. Conversely, Cyclosporin (OR: 0.13; 95% CI: 0.01–0.58; P<0.001), AZA (Imuran) (OR: 0.35; 95% CI: 0.16–0.66; P<0.001), and Zenapax (OR: 0.63; 95% CI: 0.44–0.87; P<0.001) were associated with a reduced risk of AR (**Table 2**; **Supplementary Figure S5**).

### 3.7 Intestine transplant

#### 3.7.1 Impact of immunosuppressants on patient survival

In IT recipients, patients receiving sirolimus (Rapamune) and basiliximab (Simulect) had significantly increased post transplantation survival rates compared with those in the group not receiving these medications (**Supplementary Figures S2A, C**; all P<0.05). However, thymoglobulin, alemtuzumab (Campath), rituximab (Rituxan), and generic MMF were associated with significantly decreased survival rates (**Supplementary Figures S2B, E–G**; all P<0.05).

#### 3.7.2 Impact of immunosuppressants on AR

In IT, OKT3 (Orthoclone, muromonab) (OR: 2.17; 95% CI: 1.42–3.25; P<0.001), alemtuzumab (Campath) (OR: 1.71; 95% CI: 1.27–2.27; P<0.001), Thymoglobulin (OR: 1.48; 95% CI: 1.18–1.84; P<0.001), and Atgam (OR: 2.24; 95% CI: 1.00–4.71; P<0.001) were associated with an increased risk of AR. Conversely, Simulect (basiliximab) (OR: 0.49; 95% CI: 0.29–0.79; P<0.001) and MMF (CellCept) (OR: 0.77; 95% CI: 0.59–1.00; P<0.001) were associated with a reduced risk of AR (**Table 2**; **Supplementary Figure S5**).

### 3.8 Heart–lung transplant

#### 3.8.1 Impact of immunosuppressants on patient survival

In HL transplant, patients receiving Cyclosporin and AZA had significant survival advantages (**Supplementary Figures S3A, D**; all P<0.05). Conversely, tacrolimus (Prograf), sirolimus (Rapamune), MMF (CellCept), Simulect (basiliximab), gengraf, generic Prograf and belatacept (Nulojix) were associated with significantly decreased survival rates (**Supplementary Figures S3B, C, E–G, I, J**; all P<0.05).

#### 3.8.2 Impact of immunosuppressants on AR

In HL transplant, patients receiving tacrolimus (Prograf) (OR: 2.20; 95% CI: 1.31–3.80; P<0.001) or MMF (CellCept) (OR: 1.83; 95% CI: 1.12–3.05; P<0.001) had a greater risk of AR. Patients receiving AZA (Imuran) (OR: 0.48; 95% CI: 0.27–0.83; P<0.001) and Cyclosporin (OR: 0.10; 95% CI: 0.01–0.45; P<0.001) had a lower risk of AR (**Table 2**; **Supplementary Figure S5**).

### 3.9 Pancreas–kidney transplant

#### 3.9.1 Impact of immunosuppressants on patient survival

In PK transplant, the use of ALG, cyclosporine (Cyclosporin), AZA (Imuran) and OKT3 (Orthoclone) significantly increased patient (**Supplementary Figures S4A, B, E, G**; all P<0.05). However, tacrolimus (Prograf), sirolimus (Rapamune), MMF (CellCept), thymoglobulin and, basiliximab (Simulect), mycophenolic acid (mycophenolic acid), Steroids, alemtuzumab (Campath), rituximab (Rituxan), generic Prograf (Generic tacrolimus) and belatacept (Nulojix) were associated with significantly decreased survival rates (**Supplementary Figures S4C, D, F, I–P**; all P<0.05). Additionally, pancreas-kidney combination transplant recipients may have a higher short-term survival rate with Sang Cy A, but their long-term survival rate will be lower (**Supplementary Figure S4H**).

#### 3.9.2 Impact of immunosuppressants on AR

In PK transplant, patients receiving rituximab (Rituxan) (OR: 3.95; 95% CI: 1.98–7.13; P<0.001), tacrolimus (Prograf) (OR: 1.43; 95% CI: 1.21–1.70; P<0.001), thymoglobulin (OR: 1.35; 95% CI: 1.16–1.56; P<0.001), Steroids (OR: 2.01; 95% CI: 1.44–2.89; P<0.001), alemtuzumab (Campath) (OR:1.57; 95% CI:1.23–1.99; P<0.001), mycophenolic acid (Myfortic) (OR: 1.37; 95% CI: 1.12–1.66; P<0.001), generic Prograf (generic tacrolimus) (OR: 1.38; 95% CI: 1.05–1.78; P<0.001) and generic CellCept (generic MMF) (OR: 1.39; 95% CI: 0.99–1.88; P<0.001) had a greater risk of AR. Patients receiving AZA (Imuran) (OR: 0.46; 95% CI: 0.34–0.59; P<0.001), Cyclosporin (OR: 0.47; 95% CI: 0.34–0.64; P<0.001), OKT3 (OR: 0.58; 95% CI: 0.43–0.75; P<0.001), Neoral (OR: 0.64; 95% CI: 0.48–0.85; P<0.001) and Sandimmune (OR: 0.35; 95% CI: 0.13–0.77; P<0.001) had a lower risk of AR (**Table 2**; **Supplementary Figure S5**).

### 3.10 Impact of immunosuppressants and AR on survival in different SOT patients

To further assess the impact of different immunosuppressants and AR on prognosis, a Cox regression analysis was conducted. The results revealed that AR was a risk factor for OS in all types of SOT (**Table 3**; **Supplementary Figure S6**).

**Table 3:** Cox regression analysis demonstrated the impact of immunosuppressants on survival.

Transplant type	Drug name	HR (95% CI)	P
LT	Cyclosporin	0.24 (0.22-0.26)	0.00E+00
	OKT3 (Orthoclone, muromonab)	0.45 (0.40-0.50)	0.00E+00
	Thymoglobulin	1.34 (1.27-1.42)	0.00E+00
	Prograf (tacrolimus)	1.33 (1.28-1.38)	0.00E+00
	Rapamune (sirolimus)	1.62 (1.50-1.74)	0.00E+00
	Imuran (azathioprine, AZA)	0.41 (0.39-0.44)	0.00E+00
	CellCept (MMF)	1.40 (1.36-1.45)	0.00E+00
	Steroids (prednisone, methylprednisolone, Solumedrol, Medrol)	0.78 (0.74-0.83)	9.99E-16
	Simulect (basiliximab)	1.20 (1.14-1.26)	7.32E-13
	Myfortic (mycophenolic acid)	1.22 (1.15-1.30)	5.03E-11
	EON (generic cyclosporine)	2.18 (1.71-2.77)	1.28E-10
	Gengraf	1.58 (1.37-1.83)	2.67E-10
	Atgam	0.62 (0.52-0.74)	5.36E-08
	Campath (alemtuzumab)	1.57 (1.32-1.87)	2.24E-07
	Sandimmune	0.71 (0.61-0.82)	2.48E-06
	Zenapax - Daclizumab	1.19 (1.10-1.30)	3.67E-05
	Zortress (everolimus)	1.65 (1.27-2.15)	1.90E-04
	Rituxan (rituximab)	1.27 (1.12-1.44)	2.23E-04
	ALG	0.44 (0.26-0.74)	1.48E-03
	Neoral	1.09 (1.03-1.16)	2.73E-03
	FTY 720	11.41 (1.61-80.98)	3.43E-03
	Astagraf XL (extended release tacrolimus)	1.38 (1.05-1.80)	2.03E-02
KT	**Drug name**	**HR (95% CI)**	**P**
	ALG	0.48 (0.44-0.52)	0.00E+00
	Cyclosporin	0.22 (0.21-0.24)	0.00E+00
	OKT3 (Orthoclone, muromonab)	0.58 (0.54-0.62)	0.00E+00
	Thymoglobulin	1.40 (1.36-1.44)	0.00E+00
	Simulect (basiliximab)	1.32 (1.28-1.36)	0.00E+00
	Myfortic (mycophenolic acid)	1.35 (1.31-1.40)	0.00E+00
	Prograf (tacrolimus)	1.41 (1.37-1.45)	0.00E+00
	Rapamune (sirolimus)	1.33 (1.26-1.40)	0.00E+00
	Imuran (azathioprine, AZA)	0.32 (0.30-0.33)	0.00E+00
	CellCept (MMF)	1.28 (1.24-1.31)	0.00E+00
	Gengraf	1.40 (1.29-1.52)	1.11E-16
	Campath (alemtuzumab)	1.18 (1.12-1.23)	6.79E-12
	Sandimmune	0.73 (0.67-0.80)	1.67E-11
	Astagraf XL (extended release tacrolimus)	1.54 (1.28-1.84)	3.18E-06
	Generic tacrolimus (generic Prograf)	1.09 (1.04-1.14)	5.51E-04
	Nulojix (belatacept)	1.36 (1.14-1.62)	6.94E-04
	Rituxan (rituximab)	1.31 (1.12-1.53)	8.33E-04
	Steroids (prednisone, methylprednisolone, Solumedrol, Medrol)	0.90 (0.84-0.96)	1.45E-03
	Generic MMF (generic CellCept)	1.09 (1.03-1.15)	1.86E-03
	EON (generic cyclosporine)	1.75 (1.19-2.57)	4.02E-03
	Leflunomide (LFL)	2.15 (1.25-3.70)	4.90E-03
	Atgam	0.91 (0.84-0.99)	2.38E-02
	Zenapax - Daclizumab	1.06 (1.01-1.11)	2.76E-02
HT	**Drug name**	**HR (95% CI)**	**P**
	Cyclosporin	0.28 (0.26-0.31)	0.00E+00
	Thymoglobulin	1.42 (1.31-1.52)	0.00E+00
	Simulect (basiliximab)	1.70 (1.58-1.82)	0.00E+00
	Prograf (tacrolimus)	1.59 (1.51-1.68)	0.00E+00
	Imuran (azathioprine, AZA)	0.34 (0.32-0.37)	0.00E+00
	CellCept (MMF)	1.90 (1.79-2.01)	0.00E+00
	Gengraf	1.58 (1.41-1.78)	2.33E-15
	Generic MMF (generic CellCept)	1.33 (1.21-1.47)	3.64E-09
	Zenapax - Daclizumab	1.34 (1.21-1.48)	1.03E-08
	Rapamune (sirolimus)	1.54 (1.31-1.81)	1.16E-07
	OKT3 (Orthoclone, muromonab)	0.77 (0.70-0.85)	1.16E-07
	Generic tacrolimus (generic Prograf)	1.25 (1.14-1.37)	2.87E-06
	EON (generic cyclosporine)	2.41 (1.63-3.58)	6.90E-06
	Sandimmune	0.79 (0.70-0.88)	3.92E-05
	Campath (alemtuzumab)	1.49 (1.18-1.89)	8.71E-04
	Rituxan (rituximab)	1.56 (1.13-2.15)	6.26E-03
	Generic cyclosporine	1.54 (1.08-2.20)	1.56E-02
LU	**Drug name**	**HR (95% CI)**	**P**
	Cyclosporin	0.24 (0.20-0.29)	0.00E+00
	Imuran (azathioprine, AZA)	0.66 (0.62-0.69)	0.00E+00
	CellCept (MMF)	1.24 (1.19-1.30)	0.00E+00
	Prograf (tacrolimus)	1.22 (1.16-1.28)	1.89E-15
	Neoral	0.83 (0.78-0.89)	1.19E-07
	Atgam	0.78 (0.71-0.86)	2.75E-07
	Sandimmune	0.73 (0.65-0.83)	7.49E-07
	Rituxan (rituximab)	1.63 (1.34-1.99)	1.32E-06
	Thymoglobulin	1.26 (1.13-1.41)	5.01E-05
	Gengraf	1.28 (1.11-1.48)	7.62E-04
	Generic MMF (generic CellCept)	1.13 (1.05-1.21)	9.45E-04
	Generic tacrolimus (generic Prograf)	1.12 (1.05-1.19)	1.06E-03
	OKT3 (Orthoclone, muromonab)	0.73 (0.58-0.93)	8.87E-03
	Nulojix (belatacept)	3.63 (1.36-9.67)	8.90E-03
	NRATG /NRATS	0.47 (0.24-0.90)	1.89E-02
	Campath (alemtuzumab)	1.12 (1.01-1.23)	2.64E-02
	IL - 1 Receptor Antagonist	0.17 (0.02-1.18)	3.86E-02
	Methotrexate (Folex PFS, Mexate-AQ, Rheumatrex)	0.27 (0.07-1.08)	4.29E-02
PT	**Drug name**	**HR (95% CI)**	**P**
	Rituxan (rituximab)	4.23 (3.23-5.53)	0.00E+00
	CellCept (MMF)	0.61 (0.53-0.71)	9.27E-11
	Myfortic (mycophenolic acid)	0.56 (0.39-0.79)	1.01E-03
	Campath (alemtuzumab)	1.41 (1.14-1.74)	1.21E-03
	Atgam	0.49 (0.30-0.81)	4.28E-03
	Cyclosporin	0.42 (0.22-0.78)	5.45E-03
	Steroids (prednisone, methylprednisolone, Solumedrol, Medrol)	1.55 (1.12-2.14)	7.72E-03
	Neoral	0.67 (0.44-1.00)	5.00E-02
IT	**Drug name**	**HR (95% CI)**	**P**
	Rituxan (rituximab)	1.48 (1.19-1.83)	4.24E-04
	Campath (alemtuzumab)	1.29 (1.03-1.61)	2.74E-02
	Generic MMF (generic CellCept)	2.44 (1.09-5.47)	2.96E-02
	Rapamune (sirolimus)	0.73 (0.54-0.98)	3.33E-02
	Thymoglobulin	1.20 (1.01-1.43)	3.41E-02
	Simulect (basiliximab)	0.69 (0.48-0.98)	3.72E-02
HL	**Drug name**	**HR (95% CI)**	**P**
	Cyclosporin	0.20 (0.09-0.40)	1.90E-06
	Imuran (azathioprine, AZA)	0.44 (0.30-0.65)	2.24E-05
	Prograf (tacrolimus)	1.81 (1.28-2.55)	6.83E-04
	Rapamune (sirolimus)	8.14 (2.01-33.02)	1.02E-03
	Gengraf	2.52 (1.35-4.67)	2.82E-03
	CellCept (MMF)	1.67 (1.18-2.36)	3.61E-03
	Myfortic (mycophenolic acid)	4.70 (1.49-14.87)	5.95E-03
	Generic tacrolimus (generic Prograf)	1.94 (1.04-3.61)	3.64E-02
	Simulect (basiliximab)	1.55 (1.01-2.37)	4.59E-02
PK	**Drug name**	**HR (95% CI)**	**P**
	CellCept (MMF)	1.55 (1.38-1.73)	4.90E-14
	Steroids (prednisone, methylprednisolone, Solumedrol, Medrol)	1.81 (1.54-2.13)	3.43E-13
	Simulect (basiliximab)	1.62 (1.38-1.89)	9.10E-10
	Campath (alemtuzumab)	1.85 (1.51-2.28)	2.80E-09
	Myfortic (mycophenolic acid)	1.67 (1.41-1.99)	3.44E-09
	ALG	0.52 (0.42-0.66)	1.39E-08
	Sang Cy A	3.93 (1.96-7.88)	3.79E-05
	Nulojix (belatacept)	7.43 (1.85-29.77)	1.04E-03
	Generic tacrolimus (generic Prograf)	1.38 (1.04-1.83)	2.68E-02

P-value less than 0.05 means statistically significant.

HR, hazard ratio.

By comparing the use of immunosuppressants in various types of transplantation, it was found that cyclosporine (Cyclosporin) significantly reduced the risk of death (HR < 1) in liver transplant (LT), heart transplant (HT), lung transplant (LU), heart-lung transplant (HL), pancreas transplant (PT) and kidney transplant (KT) patients, but had no significant effect in intestine transplant (IT) and pancreas–kidney transplant (PK) patients. Tacrolimus increased the risk of death (HR > 1) in liver transplant (LT), heart transplant (HT), lung transplant (LU), kidney transplant (KT) and heart-lung transplant (HL), patients, but had no significant effect in intestinal transplant (IT), pancreas transplant (PT) and pancreas–kidney transplant (PK) patients. Mycophenolate mofetil (MMF) significantly increased the risk of death (HR > 1) in liver transplant (LT), heart transplant (HT), lung transplant (LU), kidney transplant (KT), heart-lung transplant (HL) and pancreas–kidney transplant (PK) patients, reduced the risk of death (HR < 1) in pancreas transplant (PT), but had no significant effect in intestinal transplant (IT) Overall, the efficacy and risks of different immunosuppressants vary significantly across different transplant types, requiring individualized treatment selection based on the specific transplant type and patient condition(**Table 3**; **Supplementary Figure S6**).

## 4 Discussion

In this study, in which data from the large-scale, multicenter data from the Scientific Registry of Transplant Recipients (SRTR) database were utilized, the organ-specific efficacy profiles of distinct immunosuppressant classes and formulations (brand-name versus generic drugs) in solid organ transplantation (SOT), as well as the profound impact of AR events on graft and patient survival, were elucidated. Our findings reveal four critical advances: 1)Posttransplantation AR adversely affects the survival of patients with liver, kidney, heart, lung, pancreas, heart-lung, pancreas-kidney and intestine transplant; 2) The effects of the same immunosuppressant on overall survival varied across different types of solid organ transplant populations, for example, MMF (CellCept) significantly reduced overall survival rates in liver, kidney, heart, and lung transplant recipients, while demonstrating a survival benefit in PT recipients; 3) The effects of the same immunosuppressant on the development of post transplantation acute immune rejection also showed inconsistency across different solid organ transplant populations. This study revealed that OKT3 was associated with decreased AR risks in LT, HT, KT, and PK transplant, while increased AR risk in IT recipients; 4)Originator and generic immunosuppressants have different effects on the prognosis and AR in solid organ transplant patients, in the same organ transplantation type, the effects of originator drugs and their generic drugs may be entirely opposite; In heart transplantation (HT), patients receiving the originator cyclosporine (Cyclosporin) demonstrated significantly improved survival rates and reduced rejection risk, whereas its generic counterpart (EON/generic cyclosporine) was associated with decreased survival and increased rejection risk; In lung transplantation, the originator tacrolimus (Prograf) reduced rejection risk, while generic tacrolimus (generic Prograf) significantly increased rejection risk; These phenomena highlight that even within the same organ transplantation type, the efficacy and safety profiles of originator and generic drugs can differ substantially.

Solid organ transplant (SOT) patients who had AR had a worse survival rate, in line with other studies (28). Despite the fact that immunosuppressants are intended to lower the risk of rejection, their pharmacokinetic characteristics, toxicity, and individual patient variability may paradoxically raise that risk due to things like metabolic genetic polymorphisms, immune imbalances brought on by infections, metabolic disorders, inadequate dose adjustments, or treatment discontinuation (11–16). Precision dosage, toxicity monitoring, and innovative treatment techniques (such as targeted antibodies and immunological tolerance induction) may be used in future initiatives to balance these concerns.

### 4.1 Steroids

Corticosteroids demonstrate marked efficacy in suppressing AR post transplantation. Their mechanism of action primarily involves the formation of receptor–corticosteroid complexes, which are translocated into the nucleus to modulate the transcription of target genes associated with inflammatory and immune responses, thereby exerting dual immunosuppressive and anti-inflammatory effects (4, 29–34). In this study, corticosteroid use was associated with improved survival rates in LT recipients but with reduced survival rates in PK transplant recipients. This discrepancy may be attributed to variations in the duration of corticosteroid therapy and the timing of immunosuppressant withdrawal. Notably, prolonged corticosteroid administration has been linked to dose-dependent adverse effects, including infectious complications, new-onset diabetes mellitus, and osteoporosis, in transplant populations, paradoxically increasing mortality risks over extended follow-up periods (35, 36). Consequently, corticosteroid therapy necessitates careful risk–benefit assessment to balance its therapeutic efficacy against dose-limiting toxicities, with patient-tailored regimens optimized through comprehensive evaluation of recipient-specific comorbidities, immunological risk profiles, and dynamic monitoring of infection biomarkers.

### 4.2 Calcineurin inhibitors

Cyclosporine and tacrolimus, as cornerstone CNIs in transplant immunosuppression, demonstrate critical efficacy in preventing allograft rejection while facing persistent challenges in managing breakthrough immune-mediated complications during post transplantation care (37). CNIs are associated with several clinically significant adverse effects; therefore, vigilant monitoring and management are required. The most consequential complication is dose-dependent nephrotoxicity, which is mediated through dual mechanisms of afferent arteriolar vasoconstriction (via endothelin-1 upregulation) and direct tubular epithelial cell injury, which may compromise long-term allograft function. Additionally, CNIs induce hypertension through increased angiotensin II receptor sensitivity and sodium retention, metabolic disturbances, including hyperlipidemia—characterized by elevated low-density lipoprotein (LDL) cholesterol and triglycerides—frequently occur due to altered apolipoprotein metabolism. Proactive therapeutic drug monitoring (TDM) coupled with individualized CNI target ranges remains paramount in balancing immunosuppressive efficacy with toxicity mitigation (38–41).

Tacrolimus exhibits organ-specific paradoxical effects: The tacrolimus (Prograf) was associated with reduced survival and increased rejection risk in kidney (KT) and liver (LT) transplants. However, in lung transplantation (LU), the tacrolimus (Prograf) significantly lowered rejection risk while paradoxically reducing survival, suggesting an immunosuppression-toxicity imbalance in this organ. Generic tacrolimus formulations were consistently associated with elevated rejection risk and worse survival outcomes across all organ types. This discrepancy may stem from the significant pharmacokinetic variability of tacrolimus in patients and its association with CYP3A5 expression levels and genetic variations, highlighting the importance of personalized treatment for transplant recipients (42–44).

This study found that cyclosporine exerts protective effects AR in most solid organ transplant (SOT) recipients. Interestingly, in HT, the originator cyclosporine formulation emerged as a protective factor against AR, whereas its generic counterparts demonstrated a risk effect. This phenomenon suggests that within the same transplant type, originator and generic immunosuppressants may exert divergent clinical impacts. In the same time, the efficacy of the same immunosuppressant may also vary across transplant types: tacrolimus significantly reduced AR risk in LU recipients while elevating AR risk in KT.

### 4.3 mTOR inhibitors

mTOR inhibitors exert immunosuppressive effects by binding to FKBP to form a complex, which effectively inhibits mTOR activation. This inhibition halts cell cycle progression from the G1 phase to the S phase, thereby blocking T-cell activation and proliferation (45–49). mTOR inhibitors may improve outcomes by inhibiting B-cell proliferation and antibody production. In addition to their immunosuppressive effects, mTOR inhibitors also exhibit antitumor and antifibrotic properties, which help reduce the risk of post transplantation malignancies and slow the progression of allograft fibrosis (50–55). However, the use of mTOR inhibitors may be associated with several adverse effects: 1). Increased infection risk: mTOR inhibitors may suppress immune system function, increasing the risk of infections, particularly opportunistic infections; 2). Delayed wound healing: mTOR inhibitors may impair cellular proliferation and differentiation, leading to delayed wound healing. 3). Hyperlipidemia: mTOR inhibitors may disrupt lipid metabolism, resulting in hyperlipidemia; 4). Renal impairment: Certain mTOR inhibitors may cause nephrotoxicity (54, 56–60).

The role of mTOR inhibitors in liver transplantation remains a matter of debate, with findings from some studies suggesting increased survival rates in LT recipients, whereas other studies have revealed no significant benefits (61–64). Therefore, their use carries potential risks, necessitating individualized assessment of patient benefits versus risks.

### 4.4 Antiproliferative agents

Antiproliferative agents, indispensable immunosuppressants in organ transplantation, play a critical role in preventing rejection by inhibiting cellular proliferation and immune responses. However, their efficacy and safety profiles vary significantly across different agents, necessitating individualized selection based on transplant type, patient-specific factors, and other clinical considerations. AZA(azathioprine) improved survival in liver and kidney transplants, whereas MMF (CellCept) increased rejection risk and decreased survival in HT. These disparities may stem from differences in immune mechanisms and microenvironments across transplant types. For example, LT recipients may be more susceptible to AMR, against which AZA has stronger inhibitory effects. The availability of generic MMF and mycophenolic acid has expanded therapeutic options, although further validation of their bioequivalence and safety is required. The study findings demonstrate divergent profiles in pharmaceutical stability and immunosuppressive potency among distinct antiproliferative agents, highlighting the imperative for cautious substitution protocols and rigorous therapeutic monitoring during formulation transition periods (65, 66).

### 4.5 Polyclonal and monoclonal antibodies

As the first anti-CD3 monoclonal antibody approved for kidney transplantation, Muromonab (OKT3) significantly reduces acute cellular rejection risk by blocking T-cell receptor complex signaling. Early clinical trials demonstrated that sequential OKT3 therapy in KT and LT recipients achieved lower rates of biopsy-confirmed AR than did conventional triple therapy (prednisone + azathioprine + cyclosporine) (22% *vs.* 35%). However, the limited efficacy of OKT3 in heart transplantation may be attributed to distinct lymphocyte infiltration patterns within myocardial tissues (67–70). This study revealed that OKT3 was associated with decreased AR risks in LT, HT, KT, and PK transplant, while increased AR risk in IT recipients. This suggests that the same agent may exert opposing effects on AR across different organ transplant populations, a disparity potentially stemming from heterogeneity in T-cell thymic export dynamics and their differential responsiveness to CD3-targeted immunotherapy.

Polyclonal antibody agents exhibit marked variability in efficacy across different transplant types. For example, rabbit-derived antithymocyte globulin (ATG) showed significant advantages in heart transplantation, with recipients achieving a 1-year graft survival rate of 87% (*vs.* 79% in the IL-2R antagonist group). ATG significantly reduced the incidence of chronic allograft vasculopathy by modulating the regulatory T cell (Treg)/T helper 17 (Th17) balance (71, 72). In this study, the use of ATG (Atgam) in lung transplant recipients was associated with significantly improved survival rates, possibly by maintaining the innate immune balance in the lungs. Similarly, anti-lymphocyte globulin (ALG) showed a protective effect in liver transplantation, correlating with significantly improved recipient survival, which may be related to the synergistic effect of classic immunosuppressive regimens.

### 4.6 Alkylating agents

Cyclophosphamide (Cytoxan) serves as salvage therapy for refractory AR in liver transplantation, potentially increasing short-term graft survival through increased immunosuppression. However, in heart transplantation, evidence of its efficacy remains limited, with no significant reduction in rejection risk or improvement in patient survival (73).

The mechanisms by which cyclophosphamide induces immune tolerance primarily include (1) selective depletion of activated alloreactive T cells, thereby attenuating donor-specific immune attacks; (2) a reduction in the alloreactive T-cell repertoire via peripheral clonal deletion rather than intrathymic mechanisms; and (3) potential indirect enhancement of Treg functionality through immune microenvironment modulation, although further validation of its role in heart transplantation is required (74–77).

Further in-depth investigations into the precise mechanisms of action and therapeutic efficacy across different transplant types are required to elucidate the underlying pathways and clinical applicability. Such studies are crucial for guiding evidence-based clinical practice, optimizing immunosuppressive protocols, minimizing rejection episodes, and ultimately improving transplant recipients’ outcomes.

### 4.7 Novel immunosuppressants

Belatacept (Nulojix) is a fusion protein composed of the Fc fragment of human IgG1 linked to the extracellular domain of cytotoxic T-lymphocyte-associated antigen 4 (CTLA-4), which selectively inhibits T-cell activation by blocking the CD28-B7 costimulatory pathway (78). Although Belatacept (Nulojix) can have a certain effect on the immune rejection response in SOT patients, its side effects and pharmacokinetic factors may not have a significant positive impact on the prognosis of SOT patients. Researches show that Belatacept (Nulojix) is associated with adverse effects including anemia, leukopenia, hypertension, and infection risks (e.g., urinary tract infections and respiratory tract infections) (79). In this study, belatacept (Nulojix) was associated with reduced patient survival rates in lung transplantation and simultaneous pancreas-kidney transplantation. As this study did not conduct subgroup analyses on the rejection risk of belatacept, the optimization of its immunosuppressive efficacy requires further exploration in future prospective studies.

Although this study offers important insights, there are a few limitations that should be noted. First, our research may have limited our knowledge of holistic treatment methods since it was primarily concerned with comparing individual immunosuppressants rather than thoroughly evaluating the possible advantages of combination therapy. Furthermore, the retrospective methodology limited a thorough understanding of the etiology of rejection by preventing a thorough investigation of the pathophysiological pathways driving immune rejection. Last but not least, immunosuppressants were roughly classified as “used” or “not used,” without considering precise timing, duration, or dose modifications—factors that might have a substantial impact on effectiveness and safety results.

Immunosuppressants play a crucial role in solid organ transplantation, but long-term use carries safety risks, mainly including increased incidence of infections and tumors. Immunosuppression reduces the body’s defense, significantly increasing the risk of bacterial, viral, and fungal infections, with respiratory infections being particularly common. During the COVID-19 pandemic, the severity of infections and hospitalization rates among immunosuppressed patients were higher than those in the general population. Specific drugs such as mycophenolate are associated with higher infection risks, indicating that the safety profiles of different drugs vary. Furthermore, long-term immunosuppression is closely linked to the development of malignant tumors, with transplant recipients having a significantly increased risk of skin cancer, mainly due to impaired immune surveillance. At the same time, immunosuppressants promote tumor progression by altering the tumor microenvironment, making tumor risk a long-term management challenge that requires careful balancing of treatment based on individual patient conditions (80). Currently, most studies are retrospective, lacking long-term follow-up and comparisons of risks among different drugs. Future research should conduct prospective multicenter studies to clarify the safety mechanisms, balance efficacy with risks. Meanwhile, exploring local drug delivery and personalized immunosuppression strategies can help reduce systemic side effects and improve patient outcomes (81). In addition, although the SRTR database used in this study covers a large amount of clinical data on transplant patients, data missingness and incompleteness are common throughout the database. Some key variables have a high rate of missing data, posing significant challenges for data cleaning and subsequent statistical analysis. Despite our implementation of a rigorous data processing workflow and multiple validation methods to minimize data bias, data missingness may still have some impact on the robustness and representativeness of the results. Therefore, the study conclusions should be interpreted with caution in light of this limitation. Future research should focus on optimizing data collection and quality control to enhance the completeness and applicability of the database. In conclusion, the long-term safety of immunosuppressants, especially in infection prevention and tumor monitoring, still requires in-depth research to optimize the long-term survival and quality of life for transplant recipients.

Our comprehensive analysis of the SRTR database reveals that acute rejection (AR) consistently impairs survival across all solid organ transplant types, underscoring the critical need for vigilant monitoring and prevention strategies. Immunosuppressants demonstrate organ-specific and formulation-dependent effects on AR risk and patient outcomes, with originator drugs often conferring superior protection (e.g., reduced AR odds with originator tacrolimus in lung transplantation) compared to generics, which may elevate risks in certain contexts (e.g., increased mortality with generic cyclosporine in heart transplantation). These findings advocate for personalized immunosuppression regimens, integrating transplant type, patient comorbidities, and drug bioavailability considerations to optimize long-term survival. From a policy perspective, our results highlight the importance of evidence-based guidelines for medical insurance coverage. Prioritizing originator immunosuppressants in high-risk scenarios—such as heart or lung transplants—could mitigate AR and improve outcomes, potentially reducing downstream healthcare costs from rejection episodes and retransplantation. Conversely, generics may be suitable for stable, low-risk patients (e.g., certain kidney transplants) to enhance affordability without compromising efficacy. Regulatory bodies and payers should incorporate such organ-specific data into reimbursement policies, fostering equitable access while balancing cost-effectiveness. Ultimately, this study informs multidisciplinary approaches to transplantation, promoting precision medicine and public health equity in organ recipient care.

## Data Availability

The raw data supporting the conclusions of this article will be made available by the authors, without undue reservation.

## Supplementary material

Supplementary Figure 1 The influence of various immunosuppressants on survival in pancreas transplant patients. *P*-value less than 0.05 was considered significant. **(A)** The influence of Cyclosporin on survival in pancreas transplant patients. **(B)** The influence of Neoral on survival in pancreas transplant patients. **(C)** The influence of CellCept (MMF) on survival in pancreas transplant patients. **(D)** The influence of Atgam on survival in pancreas transplant patients. **(E)** The influence of Myfortic (mycophenolic acid) on survival in pancreas transplant patients. **(F)** The influence of Steroids on survival in pancreas transplant patients. **(G)** The influence of Campath (alemtuzumab) on survival in pancreas transplant patients. **(H)** The influence of Rituxan (rituximab) on survival in pancreas transplant patients.

Supplementary Figure 2 The influence of various immunosuppressants on survival in intestine transplant patients. *P*-value less than 0.05 was considered significant. **(A)** The influence of Rapamune (sirolimus) on survival in intestine transplant patients. **(B)** The influence of Thymoglobulin on survival in intestine transplant patients. **(C)** The influence of Simulect (basiliximab) on survival in intestine transplant patients. **(D)** The influence of Zortress (everolimus) on survival in intestine transplant patients. **(E)** The influence of Campath (alemtuzumab) on survival in intestine transplant patients. **(F)** The influence of Rituxan (rituximab) on survival in intestine transplant patients. **(G)** The influence of Generic MMF (generic CellCept) on survival in intestine transplant patients.

Supplementary Figure 3 The influence of various immunosuppressants on survival in heart-lung transplant patients. *P*-value less than 0.05 was considered significant. **(A)** The influence of Cyclosporin on survival in heart-lung transplant patients. **(B)** The influence of Prograf (tacrolimus) on survival in heart-lung transplant patients. **(C)** The influence of Rapamune (sirolimus) on survival in heart-lung transplant patients. **(D)** The influence of Imuran (azathioprine, AZA) on survival in heart-lung transplant patients. **(E)** The influence of CellCept (MMF) on survival in heart-lung transplant patients. **(F)** The influence of Simulect (basiliximab) on survival in heart-lung transplant patients. **(G)** The influence of Gengraf on survival in heart-lung transplant patients. **(H)** The influence of Myfortic (mycophenolic acid) on survival in heart-lung transplant patients. **(I)** The influence of Generic tacrolimus (generic Prograf) on survival in heart-lung transplant patients. **(J)** The influence of Nulojix (belatacept) on survival in heart-lung transplant patients.

Supplementary Figure 4 The influence of various immunosuppressants on survival in pancreas-kidney transplant patients. *P*-value less than 0.05 was considered significant. **(A)** The influence of ALG on survival in pancreas-kidney transplant patients. **(B)** The influence of Cyclosporin on survival in pancreas-kidney transplant patients. **(C)** The influence of Prograf (tacrolimus) on survival in pancreas-kidney transplant patients. **(D)** The influence of Rapamune (sirolimus) on survival in pancreas-kidney transplant patients. **(E)** The influence of Imuran (azathioprine, AZA) on survival in pancreas-kidney transplant patients. **(F)** The influence of CellCept (MMF) on survival in pancreas-kidney transplant patients. **(G)** The influence of OKT3 (Orthoclone, muromonab) on survival in pancreas-kidney transplant patients. **(H)** The influence of Sang Cy A on survival in pancreas-kidney transplant patients. **(I)** The influence of Thymoglobulin on survival in pancreas-kidney transplant patients. **(J)** The influence of Simulect (basiliximab) on survival in pancreas-kidney transplant patients. **(K)** The influence of Myfortic (mycophenolic acid) on survival in pancreas-kidney transplant patients. **(L)** The influence of Steroids on survival in pancreas-kidney transplant patients. **(M)** The influence of Campath (alemtuzumab) on survival in pancreas-kidney transplant patients. **(N)** The influence of Rituxan (rituximab) on survival in pancreas-kidney transplant patients. **(O)** The influence of Generic tacrolimus (generic Prograf) on survival in pancreas-kidney transplant patients. **(P)** The influence of Nulojix (belatacept) on survival in pancreas-kidney transplant patients.

Supplementary Figure 5 Forest plot demonstrating the impact of immunosuppressants on AR on logistic regression analysis. **(A)** logistic regression analysis of the effects of immunosuppressants and AR on survival in liver transplant recipients. **(B)** logistic regression analysis of the effects of immunosuppressants and AR on survival in lung transplant recipients. **(C)** logistic regression analysis of the effects of immunosuppressants and AR on survival in pancreas transplant. **(D)** logistic regression analysis of the effects of immunosuppressants and AR on survival in heart transplant recipients. **(E)** logistic regression analysis of the effects of immunosuppressants and AR on survival in kidney transplant recipients. **(F)** logistic regression analysis of the effects of immunosuppressants and AR on survival in pancreas-kidney transplant recipients. **(G)** logistic regression analysis of the effects of immunosuppressants and AR on survival in heart-lung transplant recipients. **(H)** logistic regression analysis of the effects of immunosuppressants and AR on survival in intestine transplant recipients. (OR indicates Odds Ratio; CI indicates confidence interval.)

Supplementary Figure 6 Forest plot demonstrating the impact of immunosuppressants and AR on survival based on Cox regression analysis. **(A)** Cox regression analysis of the effects of immunosuppressants and AR on survival in liver transplant recipients. **(B)** Cox regression analysis of the effects of immunosuppressants and AR on survival in lung transplant recipients. **(C)** Cox regression analysis of the effects of immunosuppressants and AR on survival in kidney transplant recipients. **(D)** Cox regression analysis of the effects of immunosuppressants and AR on survival in heart transplant recipients. **(E)** Cox regression analysis of the effects of immunosuppressants and AR on survival in pancreas-kidney transplant recipients. **(F)** Cox regression analysis of the effects of immunosuppressants and AR on survival in heart-lung transplant recipients. **(G)** Cox regression analysis of the effects of immunosuppressants and AR on survival in pancreas transplant recipients. **(H)** Cox regression analysis of the effects of immunosuppressants and AR on survival in intestinal transplant recipients. (HR indicates Hazard Ratio; CI indicates confidence interval).
