# Supplementary figures and images for: The impact of different immunosuppressants and acute immune rejection on clinical outcomes in diverse solid organ transplant recipients
Source: Front Immunol. 2026 Jan 30;16:1739468. doi: 10.3389/fimmu.2025.1739468 (PMC12903133; doi:10.3389/fimmu.2025.1739468)

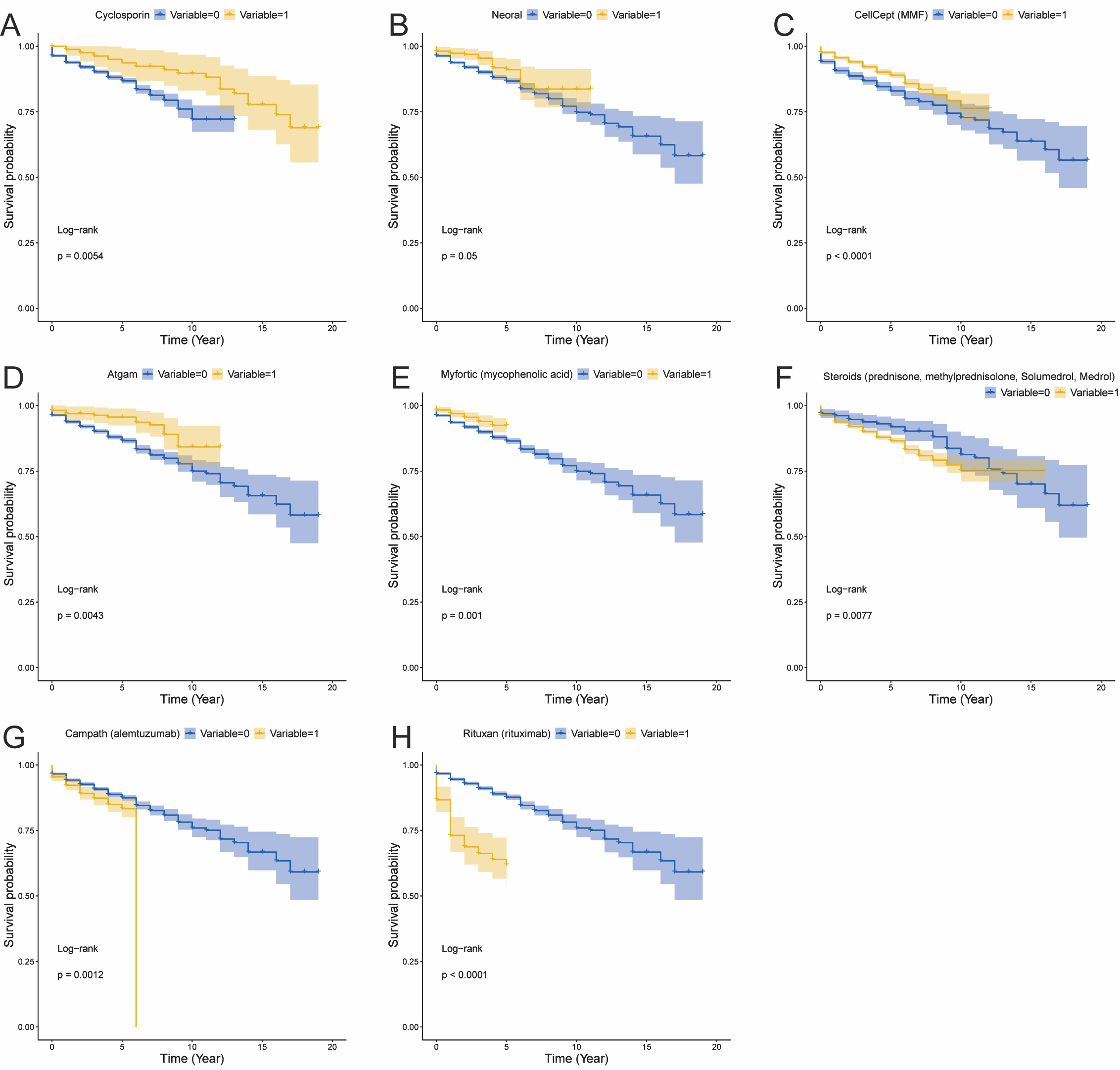

Supplement: Supplementary Figure 1 — The influence of various immunosuppressants on survival in pancreas transplant patients. P-value less than 0.05 was considered significant. (A) The influence of Cyclosporin on survival in pancreas transplant patients. (B) The influence of Neoral on survival in pancreas transplant patients. (C) The influence of CellCept (MMF) on survival in pancreas transplant patients. (D) The influence of Atgam on survival in pancreas transplant patients. (E) The influence of Myfortic (mycophenolic acid) on survival in pancreas transplant patients. (F) The influence of Steroids on survival in pancreas transplant patients. (G) The influence of Campath (alemtuzumab) on survival in pancreas transplant patients. (H) The influence of Rituxan (rituximab) on survival in pancreas transplant patients. [file Image1.tiff]

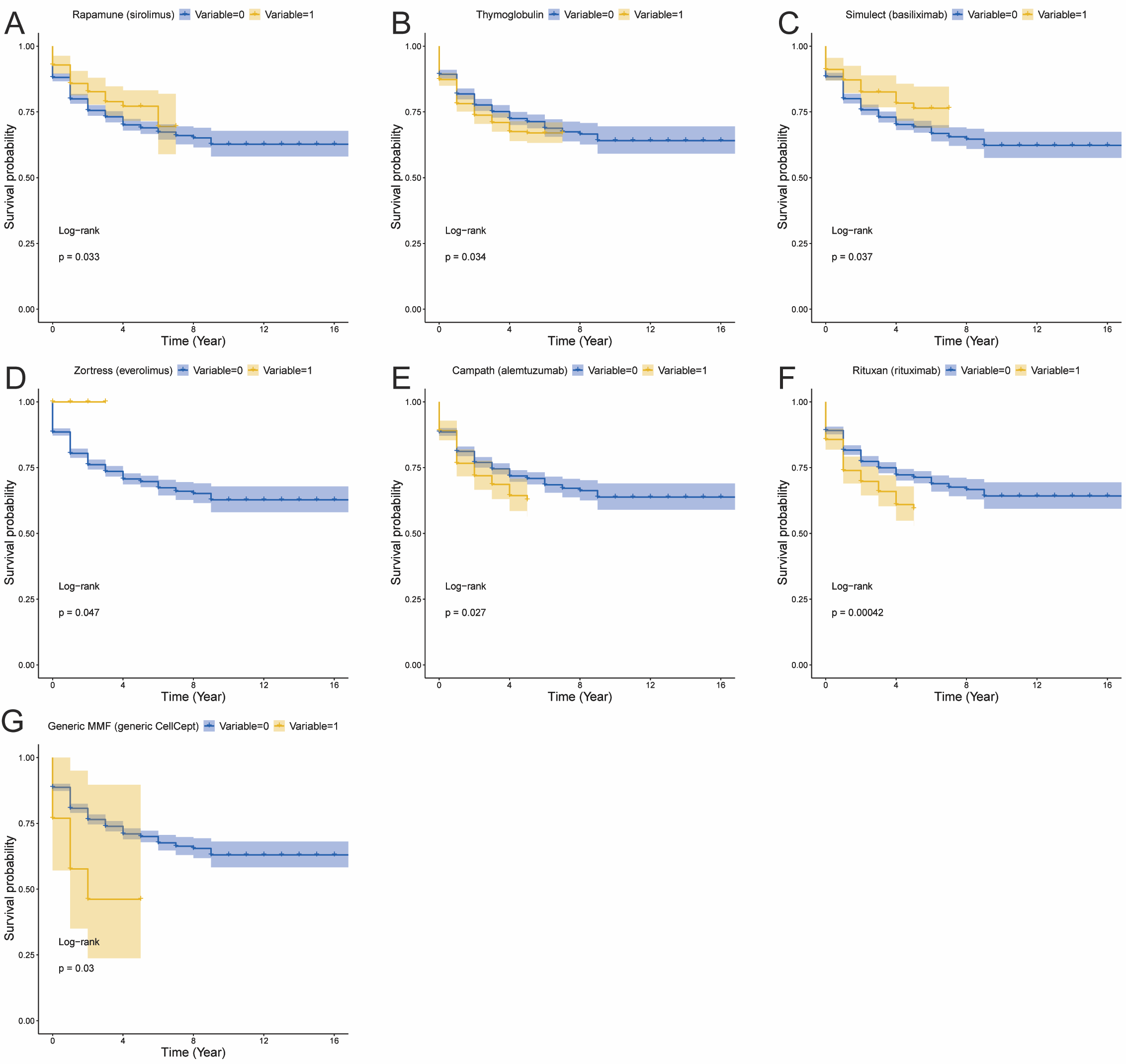

Supplement: Supplementary Figure 2 — The influence of various immunosuppressants on survival in intestine transplant patients. P-value less than 0.05 was considered significant. (A) The influence of Rapamune (sirolimus) on survival in intestine transplant patients. (B) The influence of Thymoglobulin on survival in intestine transplant patients. (C) The influence of Simulect (basiliximab) on survival in intestine transplant patients. (D) The influence of Zortress (everolimus) on survival in intestine transplant patients. (E) The influence of Campath (alemtuzumab) on survival in intestine transplant patients. (F) The influence of Rituxan (rituximab) on survival in intestine transplant patients. (G) The influence of Generic MMF (generic CellCept) on survival in intestine transplant patients. [file Image2.tiff]

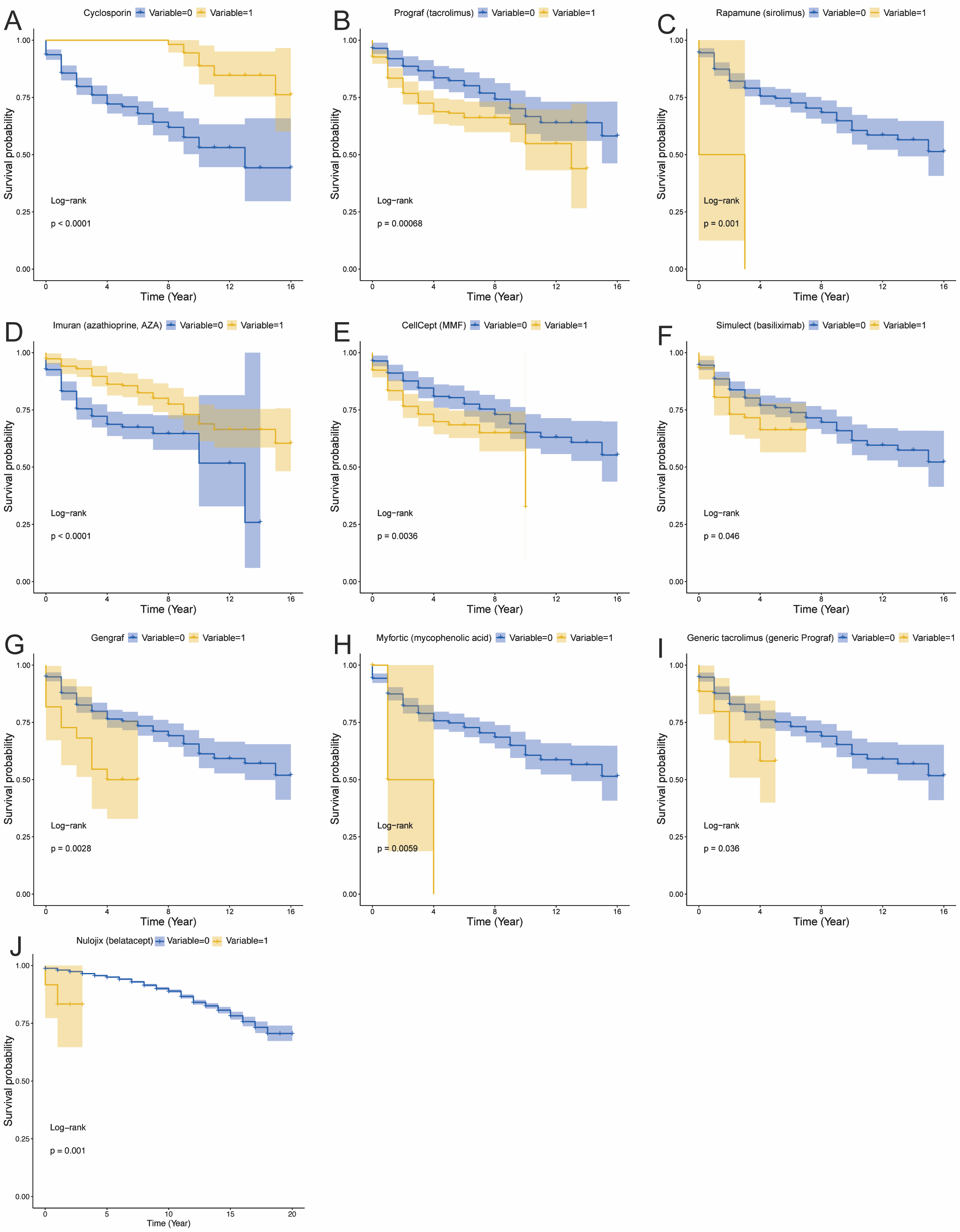

Supplement: Supplementary Figure 3 — The influence of various immunosuppressants on survival in heart-lung transplant patients. P-value less than 0.05 was considered significant. (A) The influence of Cyclosporin on survival in heart-lung transplant patients. (B) The influence of Prograf (tacrolimus) on survival in heart-lung transplant patients. (C) The influence of Rapamune (sirolimus) on survival in heart-lung transplant patients. (D) The influence of Imuran (azathioprine, AZA) on survival in heart-lung transplant patients. (E) The influence of CellCept (MMF) on survival in heart-lung transplant patients. (F) The influence of Simulect (basiliximab) on survival in heart-lung transplant patients. (G) The influence of Gengraf on survival in heart-lung transplant patients. (H) The influence of Myfortic (mycophenolic acid) on survival in heart-lung transplant patients. (I) The influence of Generic tacrolimus (generic Prograf) on survival in heart-lung transplant patients. (J) The influence of Nulojix (belatacept) on survival in heart-lung transplant patients. [file Image3.tiff]

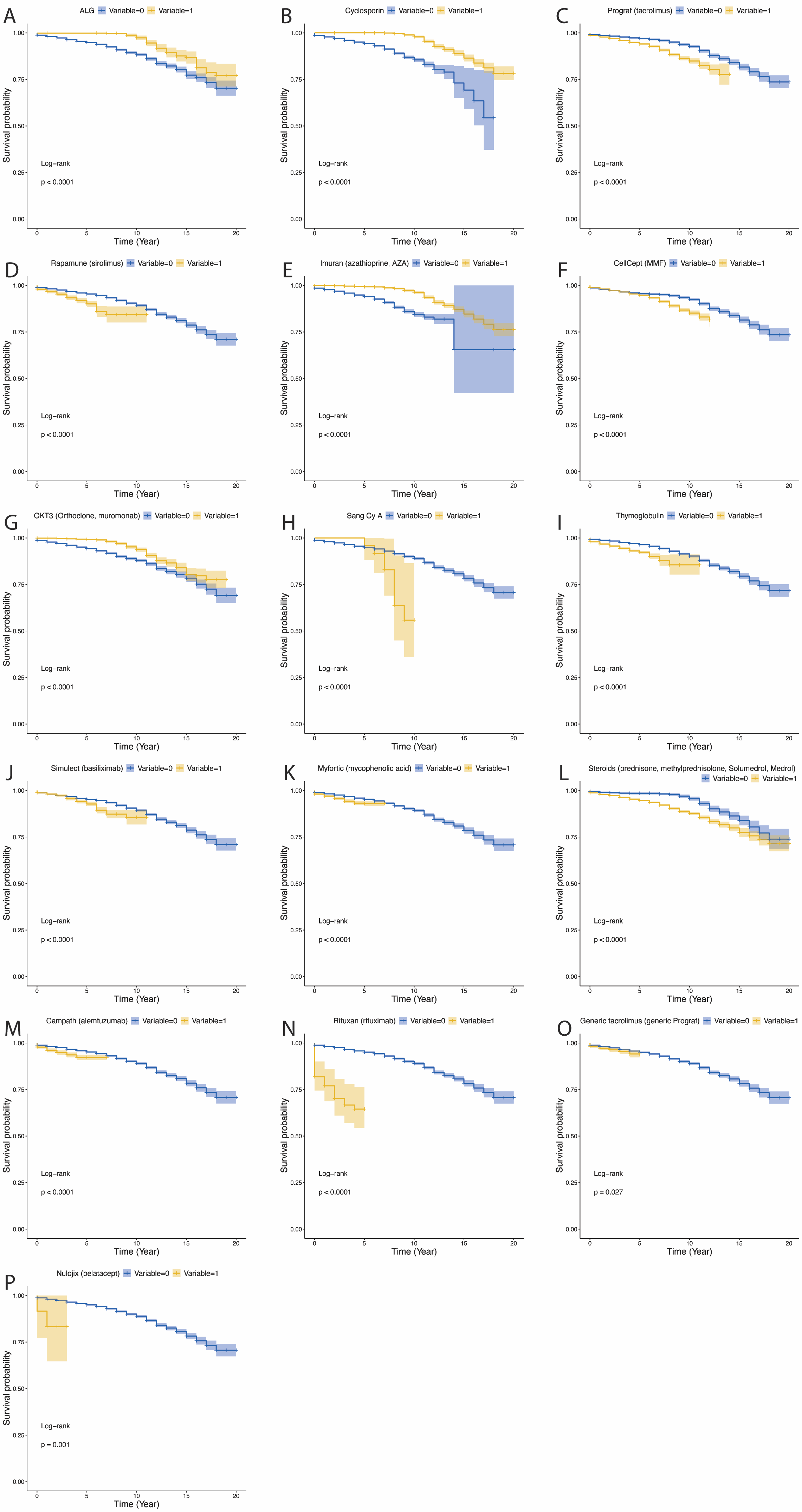

Supplement: Supplementary Figure 4 — The influence of various immunosuppressants on survival in pancreas-kidney transplant patients. P-value less than 0.05 was considered significant. (A) The influence of ALG on survival in pancreas-kidney transplant patients. (B) The influence of Cyclosporin on survival in pancreas-kidney transplant patients. (C) The influence of Prograf (tacrolimus) on survival in pancreas-kidney transplant patients. (D) The influence of Rapamune (sirolimus) on survival in pancreas-kidney transplant patients. (E) The influence of Imuran (azathioprine, AZA) on survival in pancreas-kidney transplant patients. (F) The influence of CellCept (MMF) on survival in pancreas-kidney transplant patients. (G) The influence of OKT3 (Orthoclone, muromonab) on survival in pancreas-kidney transplant patients. (H) The influence of Sang Cy A on survival in pancreas-kidney transplant patients. (I) The influence of Thymoglobulin on survival in pancreas-kidney transplant patients. (J) The influence of Simulect (basiliximab) on survival in pancreas-kidney transplant patients. (K) The influence of Myfortic (mycophenolic acid) on survival in pancreas-kidney transplant patients. (L) The influence of Steroids on survival in pancreas-kidney transplant patients. (M) The influence of Campath (alemtuzumab) on survival in pancreas-kidney transplant patients. (N) The influence of Rituxan (rituximab) on survival in pancreas-kidney transplant patients. (O) The influence of Generic tacrolimus (generic Prograf) on survival in pancreas-kidney transplant patients. (P) The influence of Nulojix (belatacept) on survival in pancreas-kidney transplant patients. [file Image4.tiff]

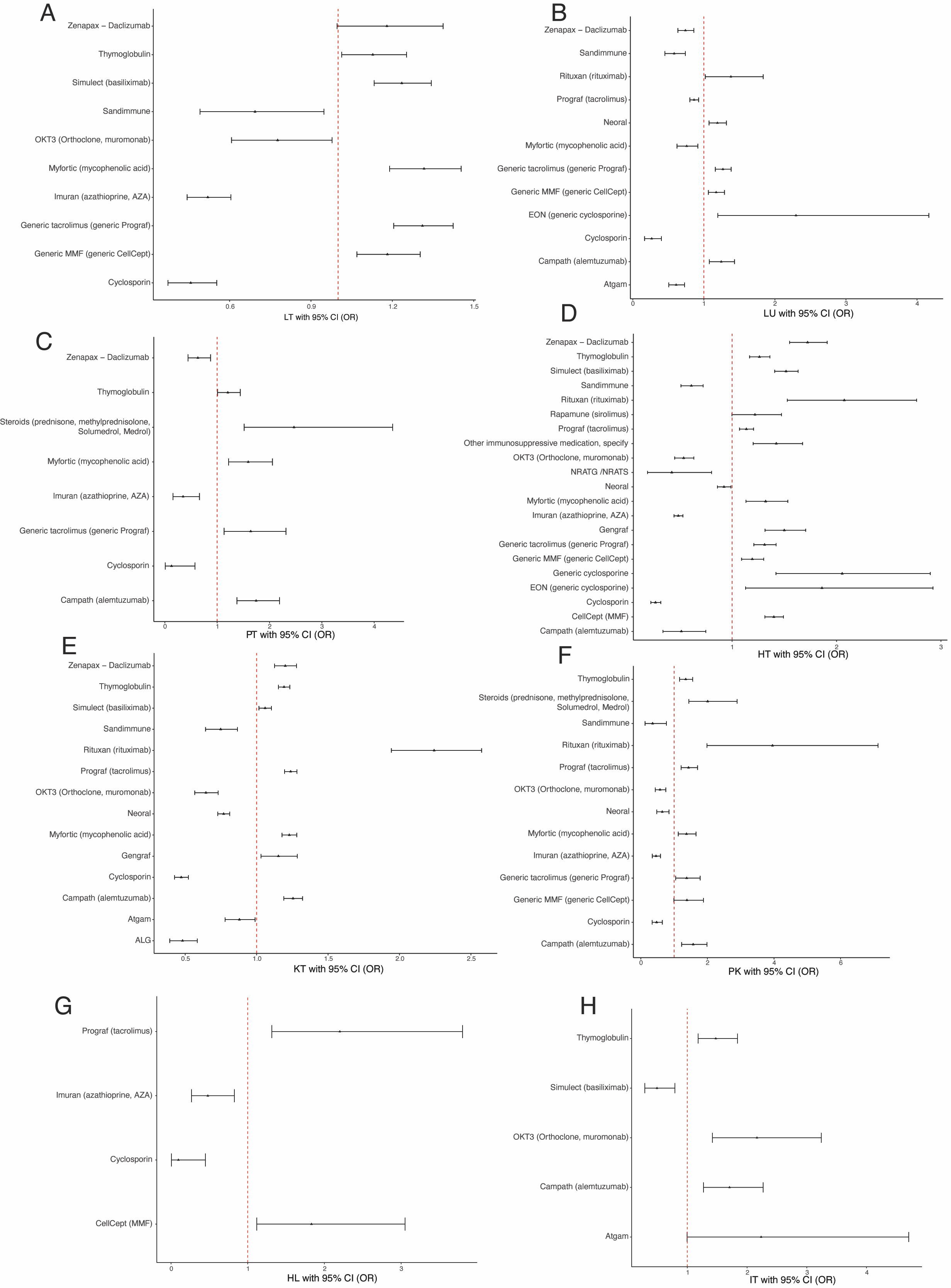

Supplement: Supplementary Figure 5 — Forest plot demonstrating the impact of immunosuppressants on AR on logistic regression analysis. (A) logistic regression analysis of the effects of immunosuppressants and AR on survival in liver transplant recipients. (B) logistic regression analysis of the effects of immunosuppressants and AR on survival in lung transplant recipients. (C) logistic regression analysis of the effects of immunosuppressants and AR on survival in pancreas transplant. (D) logistic regression analysis of the effects of immunosuppressants and AR on survival in heart transplant recipients. (E) logistic regression analysis of the effects of immunosuppressants and AR on survival in kidney transplant recipients. (F) logistic regression analysis of the effects of immunosuppressants and AR on survival in pancreas-kidney transplant recipients. (G) logistic regression analysis of the effects of immunosuppressants and AR on survival in heart-lung transplant recipients. (H) logistic regression analysis of the effects of immunosuppressants and AR on survival in intestine transplant recipients. (OR indicates Odds Ratio; CI indicates confidence interval.) [file Image5.tiff]

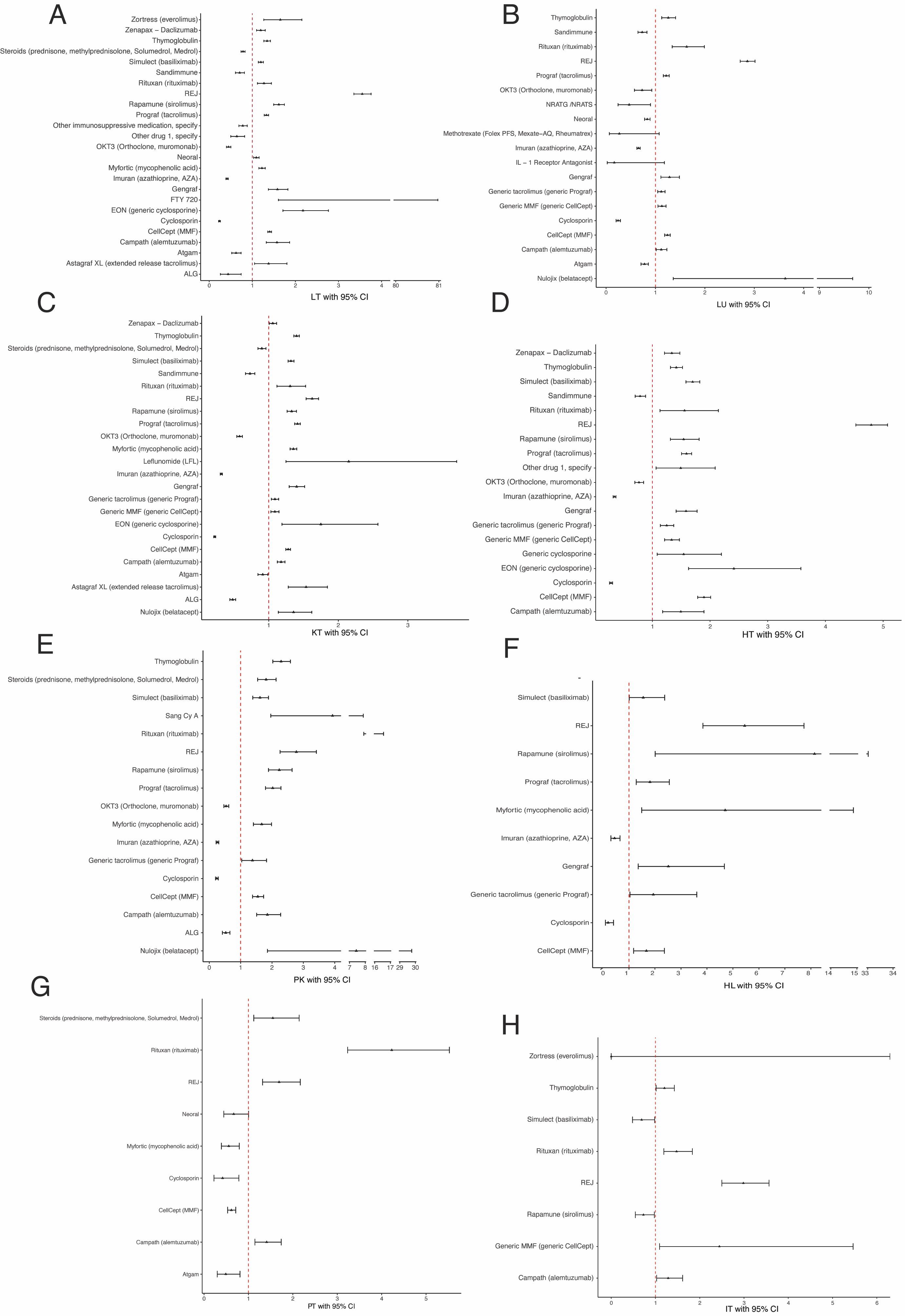

Supplement: Supplementary Figure 6 — Forest plot demonstrating the impact of immunosuppressants and AR on survival based on Cox regression analysis. (A) Cox regression analysis of the effects of immunosuppressants and AR on survival in liver transplant recipients. (B) Cox regression analysis of the effects of immunosuppressants and AR on survival in lung transplant recipients. (C) Cox regression analysis of the effects of immunosuppressants and AR on survival in kidney transplant recipients. (D) Cox regression analysis of the effects of immunosuppressants and AR on survival in heart transplant recipients. (E) Cox regression analysis of the effects of immunosuppressants and AR on survival in pancreas-kidney transplant recipients. (F) Cox regression analysis of the effects of immunosuppressants and AR on survival in heart-lung transplant recipients. (G) Cox regression analysis of the effects of immunosuppressants and AR on survival in pancreas transplant recipients. (H) Cox regression analysis of the effects of immunosuppressants and AR on survival in intestinal transplant recipients. (HR indicates Hazard Ratio; CI indicates confidence interval). [file Image6.tiff]
